# Analyzing multiple types of discrimination using implicit and explicit measures, comparing target vs. Dominant groups, in a study of smoking/vaping among community health center members in Boston, Massachusetts (2020–2022)

**DOI:** 10.1186/s12939-025-02456-9

**Published:** 2025-04-22

**Authors:** Sari L. Reisner, Nykesha Johnson, Jarvis T. Chen, Maddalena Marini, Merrily E. LeBlanc, Kenneth H. Mayer, Apriani Oendari, Donna M. Bright, Sharon Callender, Guale Valdez, Tanveer Khan, Nancy Krieger

**Affiliations:** 1 Department of Epidemiology, University of Michigan School of Public Health, Ann Arbor, MI USA; 2 Department of Epidemiology, Harvard T.H. Chan School of Public Health, Boston, MA USA; 3 The Fenway Institute, Fenway Health, Boston, MA USA; 4 Department of Social and Behavioral Sciences, Harvard T.H. Chan School of Public Health, Boston, MA USA; 5 Department of Psychology, University of Campania Luigi Vanvitelli, Caserta, CE Italy; 6 Department of Sociology and Anthropology, Northeastern University, Boston, MA USA; 7 Department of Medicine, Beth Israel Deaconess Medical Center, Harvard Medical School, Boston, MA USA; 8 Center for Community Health Education, Research and Service, Northeastern University, Boston, MA USA; 9 Mattapan Community Health Center, Boston, MA USA; 10 Harvard Street Neighborhood Health Center, Boston, MA USA

**Keywords:** Discrimination, Community health center, Methods, Health inequities, Racism, Sexism, Heterosexism, Transphobia, Cissexism, Ageism, Sizeism, Fat phobia, Implicit association test

## Abstract

**Background:**

In the United States (U.S.), the physical and mental health sequelae of diverse types of discrimination are far-reaching, severe, and contribute to population health inequities, with this work informing research on discrimination and health in both the Global North and Global South. To date, limited population health research has examined the joint impacts of discrimination measures that are explicit (i.e., self-report) and implicit (i.e., automatic mental representations), both singly and for multiple types of discrimination.

**Methods:**

Between May 28, 2020-August 4, 2022, we conducted Life + Health, a cross-sectional population-based study regarding six types of discrimination—racism, sexism, heterosexism, cissexism, ageism, and sizeism—with 699 participants (US-born, ages 25–64) from three community health centers in Boston, Massachusetts. Participants completed a Brief Implicit Association Test (B-IAT) and self-reported survey. Spearman’s correlation coefficient was estimated to assess the strength and direction of discrimination types across target/dominant groups; logistic regression models were fit to assess the association of each type of discrimination with smoking/vaping following by random-effects meta-regression modeling to pool effects across discrimination types.

**Results:**

Mean age was 37.9 years (SD = 11.2 years). Overall, 31.6% were people of color; 31.8% identified as transgender or nonbinary/genderqueer; 68.6% were sexual minority. For education, 20.5% had some college/vocational school or no college. Current cigarette/vaping was reported by 15.4% of the study population. Implicit and explicit measures were generally correlated with one another, but associations varied across discrimination types and for target/dominant groups. In random-effects meta-regression modeling, explicit compared to implicit discrimination measures were associated with a 1.18 (95% CI = 1.00-1.39) greater odds of smoking/vaping among dominant group members, but no such difference was observed among target group members.

**Conclusion:**

Implicit and explicit discrimination measures yielded distinct yet complementary insights, highlighting the importance of both. Meta-regression provided evidence of health impacts across discrimination types. Future research on discrimination and health, in diverse country contexts, should consider using both implicit and explicit measures to analyze health impacts across multiple types of discrimination.

**Supplementary Information:**

The online version contains supplementary material available at 10.1186/s12939-025-02456-9.

## Introduction

In the United States (U.S.), the physical and mental health sequelae of diverse types of discrimination are far-reaching, severe, and contribute to population health inequities (for reviews see [1, 2]), with this work informing research on discrimination and health in both the Global North and Global South. Exposure to discrimination, which systemically privileges dominant groups and is directed against the targeted socially non-dominant groups [3, 4, 5], is associated with an increased risk of poor general self-rated health and physical health [6], cardiovascular-related risks (e.g., high Body Mass Index [BMI], blood pressure) [7–9], psychological distress and anxiety [6, 10, 11], poor sleep health [12], and harmful coping behaviors including cigarette smoking and e-cigarette use [13–17]. Target groups experiencing racism, sexism, heterosexism, cissexism, ageism, and sizeism [1, 2, 18] respectively include: Black, Indigenous, and other people of color [2, 19], women [20], sexual minority (lesbian, gay, bisexual, and other non-heterosexual; LGBQ+) individuals [21, 22], transgender and nonbinary people [21, 23], people of older ages [24], and individuals who are overweight or obese [25, 26]. Many, but not all, of these targeted groups—non-Hispanic people of color in “Other race” groups, LGBQ + individuals, transgender and nonbinary people—also have a higher prevalence of cigarette smoking and/or e-cigarette use (hereafter smoking/vaping) compared to dominant groups [28–32].

Studies quantifying the association and impact of discrimination on health most commonly utilize explicit measures (i.e., self-report) which capture intentional and controlled cognitive evaluations [3, 6, 33, 34]. Yet, self-reported explicit discrimination measures are prone to social desirability bias and also, among targets of discrimination, additional underreporting due to fear of retribution, appearing vulnerable, or confirming negative stereotypes of membership in marginalized social groups (i.e., stereotype threat) [35]. Metrics for exposure to discrimination and social group preference (for target vs. dominant groups) that employ the validated Implicit Association Test (IAT) [36], may overcome the limitations of explicit measures as they rely on automatic mental representations rather than on controlled self-reported assessments of discrimination, and are less subject to social desirability biases.

To date, limited population health research has examined the joint impacts of explicit and implicit discrimination, both singly and for multiple types of discrimination [37–40]. IAT measures are generally lengthy (approximately 15 min per instrument), and thus not well-suited to population-based research. Offering new opportunities, including for analyses of multiple types of discrimination and social group preferences, is the recently developed Brief Implicit Association Test (B-IAT), a validated and more time-efficient measure (around two minutes per instrument) [41, 42]. Further, the broader sociopolitical environment in which discrimination occurs represents an important contextual factor not often evaluated alongside individual assessments of implicit and explicit discrimination [3]. Thus, research is needed to examine multiple types of implicit and explicit discrimination within the specific sociopolitical landscape in which it occurs.

The current study sought to (1) use the new B-IAT and validated explicit measures to characterize the distribution of implicit and explicit measures of six types of discrimination (racism, sexism, heterosexism, cissexism, ageism, and sizeism), and evaluate their associations, (2) explore differences in implicit and explicit discrimination, by target vs. dominant group, across six a priori selected groups (racialized group, sex/gender, sexual orientation, gender modality, age, and weight groups), considering social desirability bias and sociopolitical concerns, and (3) assess associations of implicit and explicit discrimination with current smoking/vaping in the context of current sociopolitical concerns. Our a priori hypotheses were:

- implicit bias, implicit recognition of discrimination, and explicit self-reported discrimination experiences would be positively correlated;
- members of target groups would be more likely to report implicit and explicit discrimination than members of dominant groups, and implicit discrimination would be recognized by members of target groups who do not report explicit discrimination;
- implicit and explicit measures of discrimination would independently associate with increased odds of current smoking/vaping, and the magnitude of these associations would differ by target vs. dominant group; and.
- higher scores for broader sociopolitical concerns would be positively associated with measures of both implicit and explicit discrimination and also with increased odds of current smoking/vaping.

## Methods

### Participants and procedures

The Life + Health Study was a cross-sectional population-based study designed to advance novel methods to measure and analyze discrimination for population health research [43]. Our study population comprised 699 participants recruited between May 28, 2020 and August 4, 2022 from three community health centers in Boston, Massachusetts: Fenway Health (FH), Mattapan Community Health Center (MCHC), and Harvard Street Neighborhood Health Center (HSNHC). These community health centers were selected to ensure diversity relevant to the types of discrimination being studied: FH serves a high number of sexual and gender minority (LGBTQ+) patients [44]; MCHC [45] and HSNHC [46] serve predominantly low-income persons of color; all three centers serve patients regardless of health insurance status, economic resources, or ability to pay. Participants were eligible if they had visited one of the health centers in the last two years; were born in the US to ensure comparability in potential exposure to discrimination in the US; and were ages 25–64 years. Stratified sampling was utilized to ensure sufficient sample sizes across the a priori selected social groups to investigate discrimination exposures.

We obtained a list of potentially eligible patients from each community health center. Depending on the contact information provided, we then emailed or mailed potential participants a study invitation to notify them that they would receive a phone call to assess interest and eligibility for the study, along with instructions about how to opt out of receiving this call. We called potential participants who did not opt out up to five times, calling at varying intervals and times (e.g., evenings, days, weekends). The overall response rate was 48.4% [47], comparable to other population studies conducted during the COVID-19 pandemic [48]. During the phone call, participants were screened for eligibility and if eligible, completed an electronic informed consent process, followed by the study protocol for participation. The study protocol entailed two online surveys: a self-reported survey administered via Qualtrics™ and a validated digital Brief Implicit Association Test. The study was designed to take 60 min to complete; upon completion (or at time of withdrawal) participants received the study incentive. The initial gift card value was $25 and was increased to $40 [47]. All study activities and procedures were approved by the Institutional Review Board at Harvard T.H. Chan School of Public Health (IRB-18-1128).

## Measures

We provide detailed technical descriptions of the measures we used in Appendix A, to complement the summaries we offer here.

*Implicit Discrimination and Preference Measures.* Implicit discrimination was measured using a separate Brief Implicit Association Test (B-IAT) [41] for: racism, sexism, heterosexism, cissexism, ageism, and sizeism. For each discrimination type, a Target/Dominant B-IAT assessed an implicit recognition of discrimination toward members of the target group, and a Good/Bad B-IAT measured internalized preference (implicit bias) for the target group. Scores could range from + 2 (implicit preference for and recognition of discrimination against target group) to − 2 (implicit preference for and recognition of discrimination against dominant group), with a 0 indicating neutrality between attributes and social categories. Each brief IAT took approximately two minutes to administer, totaling 12 min.

*Explicit Self-Reported Discrimination and Preference Measures.* The validated Experiences of Discrimination (EOD) measure assessed explicit self-reported discrimination (from “never” to “rarely to “sometimes” to “often”) in 10 domains, ranging from public venues to at home, due to their race, gender identity, sexuality, gender modality, age, and weight [49]. Explicit preferences were measured using self-reported items on a 7-point scale, spanning from strong preference for the target group to strong preference for the dominant group.

*Social Groups.* Target vs. dominant groups were operationalized corresponding to the six categories of social discrimination (see Appendix A). For sizeism, we computed body mass index (BMI; weight/height^2^) based on self-reported height and weight data, and dichotomized < 30 BMI (underweight, healthy weight, overweight) vs. >=30 BMI (obese) [50]. We also created a variable summing the number of target groups a person reported being in from 0 to 6.

*Sociopolitical Concerns.* We assessed sociopolitical concerns using a 15-item scale from the Gallup Poll Social Series [51], fielded since 2001, measuring how much people personally worry (using a 5-point scale) about each problem (from “not at all” to “a great deal”), with scores ranging from 0 to 45 [52]. Problems listed included topics related to hunger and homelessness, the environment, immigration, race relations, healthcare, and unemployment. Internal consistency reliability of the measure in this sample was high (α = 0.87).

*Social Desirability Bias.* We used the validated RAND Socially Desirable Response Set Five-Item Survey (SDRS-5) to measure social desirability bias [53]. Items were summed (0–5) and transformed to a log linear scale (0-100).

*Sociodemographic Characteristics and Context.* Self-reported sociodemographic data pertained to: individual educational attainment [54], current relationship status [49], childhood and adult economic deprivation [54, 55], occupational class [54, 56], and housing tenure [57]. To characterize participants’ residential context, we geocoded the mailing address they provided at recruitment to the census tract, and utilized the 5-year estimate (2015–2019) American Community Survey (ACS) census tract data for: composition by racialized group, Index for Concentration at the Extremes (ICE) for racialized economic segregation and housing tenure [58], and median income (2019 inflation-adjusted US dollars).

*Health Behavior: Current Smoking/Vaping.* Four items drawn from the U.S. Behavioral Risk Factor Surveillance Survey asked about lifetime and current cigarette use (smoking) and e-cigarette (vaping) [59]. We combined variables to create a binary indicator of current smoking/vaping to capture a current stress-responsive health behavior.

### Data analysis

We computed univariate descriptive statistics and visualized distributions for the overall sample and by social discrimination groups. Bivariate tests compared implicit and explicit discrimination measures across social groups using t-tests for continuous indicators, χ2 tests for categorical or binary variables and the Spearman’s product moment correlation coefficient (*r*) to assess the strength and direction of their relationships (range: −1 to 1); effect sizes were interpreted as 0.10 (weak or small effect) 0.30 (moderate effect), 0.50 (strong or large effect) [60]. We generated a separate correlation matrix for each type of discrimination, stratified by target vs. dominant group, and by self-reported explicit discrimination (EOD = 0, EOD > = 1). These matrices also included social desirability, sociopolitical concerns, and number of target group memberships.

To assess associations of implicit and explicit discrimination measures with current smoking/vaping, we fit bivariate and multivariable logistic regression models, yielding odds ratios (ORs) and 95% confidence intervals (CI). For each of the six types of discrimination, a bivariate model regressed current smoking/vaping on implicit and explicit discrimination measures, followed by a multivariable model that adjusted for all other discrimination measures, number of domains as a target group member, age, education, childhood economic deprivation, sociopolitical concerns, and social desirability, and clinical recruitment site. We then conducted a random-effects meta-regression analysis [61], pooling the results from each of these discrimination models to characterize common patterns of association for implicit vs. explicit measures of discrimination among target vs. dominant groups with respect to current smoking/vaping. To account for statistical heterogeneity across measure type (implicit vs. explicit) and target group (target vs. dominant group), the model included an interaction term (implicit vs. explicit x target vs. dominant group), and the domain of discrimination. Multiple imputation using fully conditional specification was implemented to impute missing B-IAT data [62]. All analyses were conducted in R statistical software [63].

## Results

### Sample characteristics

Table 1 presents sample characteristics overall and stratified by discrimination type (for each of the six experiments) and by target vs. dominant group. Mean age was 37.9 years (SD = 11.2 years) Overall, 31.6% were people of color; 31.8% identified as transgender or nonbinary/genderqueer; 68.6% were sexual minority. For education, 20.5% had some college/vocational school or no college. 57.4% were married or in a relationship. 36.6% reported childhood economic deprivation, 29.2% adult economic deprivation, and 28.3% food insecurity; 15.0% were unemployed or not in the paid labor force. Approximately half (51.5%) reported renting their housing. Approximately one-third (38.2%) had a self-reported BMI > = 30. The mean sociopolitical concerns score was 25.3 (SD = 8.4) (range 0–45). For further contextualization, Census tract characteristics of composition by racialized group, and also racialized economic segregation and housing tenure segregation (using the Index for Concentration at the Extremes) are presented in Table 1.

**Table 1:** Health behaviors, social characteristics and context of Life + Health study participants (US-Born ages 25–64 years recruited from 3 community health centers), stratified by target vs. dominant groups for 6 types of discrimination, Boston, Massachusetts, 2020–2022

	Types of Discrimination: a priori comparison groups (target and dominant)													
	Total Sample	Racism (***N* = 699)**		Sexism (***N* = 699)**			Heterosexism ^a^ (***N* = 697)**		Cissexism ^b^ (***N* = 699)**		Ageism (***N* = 699)**		Sizeism ^c^ (***N* = 647)**	
**Variable**	**Overall**	**White non-Hispanic**	**Person of Color**	**Man**	**Woman**	**Nonbinary/** **Genderqueer**	**LGBQ**	**Straight**	**Cisgender**	**Not Cisgender**	**25–44** **yrs old**	**45–64** **yrs old**	**“Not Obese"**	**“Obese”**
**N**	699	478 (68.4%)	221 (31.6%)	313 (44.8%)	254 (36.3%)	132 (18.9%)	478 (68.6%)	219 (31.4%)	477 (68.2%)	222 (31.8%)	520 (74.4%)	179 (25.6%)	400 (61.8%)	247 (38.2%)
**Health Behavior: Current Smoking/Vaping** ^**d**^														
**Current Smoker or Regular Vaper**	108 (15.5%)	64 (13.4%)	44 (19.9%)	45 (14.4%)	36 (14.2%)	27 (20.5%)	77 (16.1%)	30 (13.7%)	66 (13.8%)	42 (18.9%)	81 (15.6%)	27 (15.1%)	65 (16.2%)	36 (14.6%)
**Age**,** BMI**,** & Social Group Membership**^**e**^														
**Age in Years: Mean (SD)**	37.87 (11.15)	38.09 (11.50)	40.65 (12.12)	36.67 (10.58)	33.95 (8.46)	37.78 (11.00)	38.11 (11.71)	39.59 (11.94)	34.38 (8.62)	32.09 (5.07)	54.92 (5.64)	36.48 (10.65)	40.23 (11.87)	37.87 (11.15)
**BMI: Mean (SD)**	28.39 (6.61)	30.87 (8.19)	28.68 (6.41)	29.19 (7.99)	30.18 (7.44)	28.76 (6.49)	29.93 (8.57)	29.00 (7.03)	29.47 (7.63)	28.74 (7.39)	30.36 (6.58)	24.73 (2.89)	36.31 (6.34)	28.39 (6.61)
[Missing: N (%)]	[31 (6.5%)]	[21 (9.5%)]	[23 (7.3%)]	[23 (9.1%)]	[6 (4.5%)]	[35 (7.3%)]	[17 (7.8%)]	[38 (8.0%)]	[14 (6.3%)]	[37 (7.1%)]	[15 (8.4%)]	[0 (0.0%)]	[0 (0.0%)]	[31 (6.5%)]
**Racialized Group: N (%)** ^**f**^														
White Non-Hispanic Only	478 (68.4%)	478 (100.0%)	NA	221 (70.6%)	163 (64.2%)	94 (71.2%)	365 (76.4%)	113 (51.6%)	311 (65.2%)	167 (75.2%)	357 (68.7%)	121 (67.6%)	297 (74.2%)	150 (60.7%)
Black Non-Hispanic Only	107 (15.3%)	NA	107 (48.4%)	40 (12.8%)	50 (19.7%)	17 (12.9%)	38 (7.9%)	67 (30.6%)	85 (17.8%)	22 (9.9%)	65 (12.5%)	42 (23.5%)	38 (9.5%)	57 (23.1%)
Hispanic Only	30 (4.3%)	NA	30 (13.6%)	14 (4.5%)	11 (4.3%)	5 (3.8%)	18 (3.8%)	12 (5.5%)	23 (4.8%)	7 (3.2%)	25 (4.8%)	5 (2.8%)	13 (3.2%)	15 (6.1%)
Asian Only	25 (3.6%)	NA	25 (11.3%)	12 (3.8%)	11 (4.3%)	2 (1.5%)	11 (2.3%)	14 (6.4%)	20 (4.2%)	5 (2.3%)	22 (4.2%)	3 (1.7%)	19 (4.8%)	5 (2.0%)
Middle Eastern or North African Only	1 (0.1%)	NA	1 (0.5%)	0 (0.0%)	0 (0.0%)	1 (0.8%)	1 (0.2%)	0 (0.0%)	0 (0.0%)	1 (0.5%)	1 (0.2%)	0 (0.0%)	1 (0.2%)	0 (0.0%)
Two or More Racialized Groups	58 (8.3%)	NA	58 (26.2%)	26 (8.3%)	19 (7.5%)	13 (9.8%)	45 (9.4%)	13 (5.9%)	38 (8.0%)	20 (9.0%)	50 (9.6%)	8 (4.5%)	32 (8.0%)	20 (8.1%)
American Indian/Alaskan Native (AIAN) Only	0 (0.0%)	0 (0.0%)	0 (0.0%)	0 (0.0%)	0 (0.0%)	0 (0.0%)	0 (0.0%)	0 (0.0%)	0 (0.0%)	0 (0.0%)	0 (0.0%)	0 (0.0%)	0 (0.0%)	0 (0.0%)
Native Hawaiian/Pacific Islander (NHPI) Only	0 (0.0%)	0 (0.0%)	0 (0.0%)	0 (0.0%)	0 (0.0%)	0 (0.0%)	0 (0.0%)	0 (0.0%)	0 (0.0%)	0 (0.0%)	0 (0.0%)	0 (0.0%)	0 (0.0%)	0 (0.0%)
**Gender Identity: N (%)**														
Cisgender Man	264 (37.8%)	184 (38.5%)	80 (36.2%)	264 (84.3%)	NA	NA	195 (40.8%)	69 (31.5%)	264 (55.3%)	NA	157 (30.2%)	107 (59.8%)	155 (38.8%)	91 (36.8%)
Cisgender Woman	213 (30.5%)	127 (26.6%)	86 (38.9%)	NA	213 (83.9%)	NA	84 (17.6%)	129 (58.9%)	213 (44.7%)	NA	165 (31.7%)	48 (26.8%)	121 (30.2%)	72 (29.1%)
Transgender Man	49 (7.0%)	37 (7.7%)	12 (5.4%)	49 (15.7%)	NA	NA	43 (9.0%)	5 (2.3%)	NA	49 (22.1%)	45 (8.7%)	4 (2.2%)	28 (7.0%)	16 (6.5%)
Transgender Woman	41 (5.9%)	36 (7.5%)	5 (2.3%)	NA	41 (16.1%)	NA	37 (7.7%)	3 (1.4%)	NA	41 (18.5%)	35 (6.7%)	6 (3.4%)	25 (6.2%)	13 (5.3%)
Nonbinary/ Transgender Nonbinary/ Genderqueer	132 (18.9%)	94 (19.7%)	38 (17.2%)	NA	NA	132 (100.0%)	119 (24.9%)	13 (5.9%)	NA	132 (59.5%)	118 (22.7%)	14 (7.8%)	71 (17.8%)	55 (22.3%)
**Sexuality: N (%)** ^**g**^														
Straight or Heterosexual	219 (31.3%)	113 (23.6%)	106 (48.0%)	74 (23.6%)	132 (52.0%)	13 (9.8%)	NA	219 (100.0%)	198 (41.5%)	21 (9.5%)	162 (31.2%)	57 (31.8%)	118 (29.5%)	84 (34.0%)
Gay or Lesbian	222 (31.8%)	170 (35.6%)	52 (23.5%)	172 (55.0%)	40 (15.7%)	10 (7.6%)	222 (46.4%)	NA	202 (42.3%)	20 (9.0%)	134 (25.8%)	88 (49.2%)	136 (34.0%)	72 (29.1%)
Queer	98 (14.0%)	78 (16.3%)	20 (9.0%)	21 (6.7%)	20 (7.9%)	57 (43.2%)	98 (20.5%)	NA	17 (3.6%)	81 (36.5%)	90 (17.3%)	8 (4.5%)	57 (14.2%)	37 (15.0%)
Two or More Sexual Minority Identities	97 (13.9%)	76 (15.9%)	21 (9.5%)	28 (8.9%)	34 (13.4%)	35 (26.5%)	97 (20.3%)	NA	35 (7.3%)	62 (27.9%)	84 (16.2%)	13 (7.3%)	54 (13.5%)	38 (15.4%)
Bisexual	44 (6.3%)	28 (5.9%)	16 (7.2%)	15 (4.8%)	22 (8.7%)	7 (5.3%)	44 (9.2%)	NA	22 (4.6%)	22 (9.9%)	37 (7.1%)	7 (3.9%)	25 (6.2%)	9 (3.6%)
Asexual	7 (1.0%)	6 (1.3%)	1 (0.5%)	1 (0.3%)	1 (0.4%)	5 (3.8%)	7 (1.5%)	NA	0 (0.0%)	7 (3.2%)	6 (1.2%)	1 (0.6%)	4 (1.0%)	3 (1.2%)
Pansexual	7 (1.0%)	6 (1.3%)	1 (0.5%)	0 (0.0%)	3 (1.2%)	4 (3.0%)	7 (1.5%)	NA	2 (0.4%)	5 (2.3%)	6 (1.2%)	1 (0.6%)	5 (1.2%)	2 (0.8%)
Same Gender Loving	2 (0.3%)	0 (0.0%)	2 (0.9%)	1 (0.3%)	0 (0.0%)	1 (0.8%)	2 (0.4%)	NA	1 (0.2%)	1 (0.5%)	0 (0.0%)	2 (1.1%)	0 (0.0%)	0 (0.0%)
Questioning	1 (0.1%)	1 (0.2%)	0 (0.0%)	0 (0.0%)	1 (0.4%)	0 (0.0%)	1 (0.2%)	NA	0 (0.0%)	1 (0.5%)	1 (0.2%)	0 (0.0%)	1 (0.2%)	0 (0.0%)
Unspecified	2 (0.3%)	0 (0.0%)	2 (0.9%)	1 (0.3%)	1 (0.4%)	0 (0.0%)	0 (0.0%)	0 (0.0%)	0 (0.0%)	2 (0.9%)	0 (0.0%)	2 (1.1%)	0 (0.0%)	2 (0.8%)
**Educational Attainment: N (%)**														
Graduate Degree	286 (40.9%)	216 (45.2%)	70 (31.7%)	127 (40.6%)	116 (45.7%)	43 (32.6%)	196 (41.0%)	90 (41.1%)	214 (44.9%)	72 (32.4%)	216 (41.5%)	70 (39.1%)	173 (43.2%)	90 (36.4%)
Four Years of College	270 (38.6%)	197 (41.2%)	73 (33.0%)	127 (40.6%)	83 (32.7%)	60 (45.5%)	188 (39.3%)	82 (37.4%)	180 (37.7%)	90 (40.5%)	212 (40.8%)	58 (32.4%)	171 (42.8%)	86 (34.8%)
Some College/Vocational School	102 (14.6%)	52 (10.9%)	50 (22.6%)	43 (13.7%)	39 (15.4%)	20 (15.2%)	69 (14.4%)	31 (14.2%)	60 (12.6%)	42 (18.9%)	70 (13.5%)	32 (17.9%)	43 (10.8%)	50 (20.2%)
No College	41 (5.9%)	13 (2.7%)	28 (12.7%)	16 (5.1%)	16 (6.3%)	9 (6.8%)	25 (5.2%)	16 (7.3%)	23 (4.8%)	18 (8.1%)	22 (4.2%)	19 (10.6%)	13 (3.2%)	21 (8.5%)
**Current Relationship Status: N (%)**														
Married	172 (24.6%)	133 (27.8%)	39 (17.6%)	88 (28.1%)	70 (27.6%)	14 (10.6%)	108 (22.6%)	64 (29.2%)	139 (29.1%)	33 (14.9%)	108 (20.8%)	64 (35.8%)	96 (24.0%)	60 (24.3%)
In a Relationship	229 (32.8%)	153 (32.0%)	76 (34.4%)	83 (26.5%)	81 (31.9%)	65 (49.2%)	164 (34.3%)	63 (28.8%)	142 (29.8%)	87 (39.2%)	199 (38.3%)	30 (16.8%)	131 (32.8%)	85 (34.4%)
Single	235 (33.6%)	152 (31.8%)	83 (37.6%)	114 (36.4%)	80 (31.5%)	41 (31.1%)	157 (32.8%)	78 (35.6%)	160 (33.5%)	75 (33.8%)	177 (34.0%)	58 (32.4%)	139 (34.7%)	80 (32.4%)
Divorced/Separated	45 (6.4%)	30 (6.3%)	15 (6.8%)	21 (6.7%)	15 (5.9%)	9 (6.8%)	35 (7.3%)	10 (4.6%)	24 (5.0%)	21 (9.5%)	25 (4.8%)	20 (11.2%)	25 (6.2%)	15 (6.1%)
Widowed	4 (0.6%)	0 (0.0%)	4 (1.8%)	0 (0.0%)	4 (1.6%)	0 (0.0%)	1 (0.2%)	3 (1.4%)	4 (0.8%)	0 (0.0%)	1 (0.2%)	3 (1.7%)	1 (0.2%)	3 (1.2%)
Other	14 (2.0%)	10 (2.1%)	4 (1.8%)	7 (2.2%)	4 (1.6%)	3 (2.3%)	13 (2.7%)	1 (0.5%)	8 (1.7%)	6 (2.7%)	10 (1.9%)	4 (2.2%)	8 (2.0%)	4 (1.6%)
**Sociopolitical Concerns: Mean (SD)** ^**h**^	25.33 (8.39)	24.49 (7.65)	27.17 (9.55)	25.02 (8.34)	25.29 (8.67)	26.17 (7.93)	25.29 (7.65)	25.32 (9.75)	25.25 (8.49)	25.52 (8.17)	24.85 (8.15)	26.75 (8.90)	24.51 (8.06)	26.56 (8.56)
**Social Desirability Bias: Mean (SD)** ^**i**^	24.52 (25.61)	22.34 (23.52)	29.23 (29.14)	23.19 (25.82)	28.11 (26.95)	20.76 (21.46)	21.72 (24.36)	30.32 (27.11)	25.28 (26.39)	22.88 (23.82)	22.31 (23.83)	30.95 (29.31)	23.50 (25.09)	25.75 (26.46)
**Adult & childhood economic security**,** current occupational class**,** and current housing tenure**														
**Childhood Economic Deprivation: N (%)** ^**j**^	256 (36.6%)	128 (26.8%)	128 (57.9%)	110 (35.1%)	86 (33.9%)	60 (45.5%)	164 (34.3%)	91 (41.6%)	168 (35.2%)	88 (39.6%)	188 (36.2%)	68 (38.0%)	117 (29.2%)	115 (46.6%)
**Adult Economic Deprivation: N (%)** ^**k**^	204 (29.2%)	109 (22.8%)	95 (43.0%)	78 (24.9%)	71 (28.0%)	55 (41.7%)	136 (28.5%)	68 (31.1%)	120 (25.2%)	84 (37.8%)	153 (29.4%)	51 (28.5%)	89 (22.2%)	97 (39.3%)
**Adult Food Insecurity: N (%)** ^**l**^	198 (28.3%)	106 (22.2%)	92 (41.6%)	75 (24.0%)	69 (27.2%)	54 (40.9%)	130 (27.2%)	68 (31.1%)	115 (24.1%)	83 (37.4%)	155 (29.8%)	43 (24.0%)	91 (22.8%)	85 (34.4%)
**Occupational class of participant: N (%)**														
Non-supervisory Employee	330 (47.2%)	92 (41.6%)	238 (49.8%)	145 (46.3%)	122 (48.0%)	63 (47.7%)	223 (46.7%)	107 (48.9%)	224 (47.0%)	106 (47.7%)	270 (51.9%)	60 (33.5%)	206 (51.5%)	103 (41.7%)
Owner, Self-employed, or Supervisory Employee	227 (32.5%)	64 (29.0%)	163 (34.1%)	109 (34.8%)	83 (32.7%)	35 (26.5%)	169 (35.4%)	58 (26.5%)	168 (35.2%)	59 (26.6%)	157 (30.2%)	70 (39.1%)	126 (31.5%)	81 (32.8%)
Unemployed or Not in the Paid Labor Force	105 (15.0%)	44 (19.9%)	61 (12.8%)	45 (14.4%)	34 (13.4%)	26 (19.7%)	68 (14.2%)	37 (16.9%)	63 (13.2%)	42 (18.9%)	66 (12.7%)	39 (21.8%)	47 (11.7%)	48 (19.4%)
Other	32 (4.6%)	17 (7.7%)	15 (3.1%)	11 (3.5%)	15 (5.9%)	6 (4.5%)	16 (3.3%)	14 (6.4%)	19 (4.0%)	13 (5.9%)	23 (4.4%)	9 (5.0%)	19 (4.8%)	12 (4.9%)
Not Specified	5 (0.7%)	4 (1.8%)	1 (0.2%)	3 (1.0%)	0 (0.0%)	2 (1.5%)	2 (0.4%)	3 (1.4%)	3 (0.6%)	2 (0.9%)	4 (0.8%)	1 (0.6%)	2 (0.5%)	3 (1.2%)
**Housing tenure: N (%)**														
Home Owned with a Mortgage/Loan	247 (35.3%)	177 (37.0%)	70 (31.7%)	126 (40.3%)	95 (37.4%)	26 (19.7%)	174 (36.4%)	73 (33.3%)	189 (39.6%)	58 (26.1%)	155 (29.8%)	92 (51.4%)	140 (35.0%)	85 (34.4%)
Home Owned Free and Clear	39 (5.6%)	28 (5.9%)	11 (5.0%)	23 (7.3%)	12 (4.7%)	4 (3.0%)	28 (5.9%)	11 (5.0%)	33 (6.9%)	6 (2.7%)	23 (4.4%)	16 (8.9%)	26 (6.5%)	13 (5.3%)
Rent Home	360 (51.5%)	247 (51.7%)	113 (51.1%)	146 (46.6%)	122 (48.0%)	92 (69.7%)	241 (50.4%)	118 (53.9%)	222 (46.5%)	138 (62.2%)	299 (57.5%)	61 (34.1%)	206 (51.5%)	128 (51.8%)
Occupied Without Payment of Cash Rent	11 (1.6%)	6 (1.3%)	5 (2.3%)	4 (1.3%)	5 (2.0%)	2 (1.5%)	9 (1.9%)	2 (0.9%)	8 (1.7%)	3 (1.4%)	9 (1.7%)	2 (1.1%)	7 (1.8%)	4 (1.6%)
Not Specified	39 (5.6%)	18 (3.8%)	21 (9.5%)	12 (3.8%)	19 (7.5%)	8 (6.1%)	23 (4.8%)	15 (6.8%)	24 (5.0%)	15 (6.8%)	32 (6.2%)	7 (3.9%)	18 (4.5%)	17 (6.9%)
Homeless	3 (0.4%)	2 (0.4%)	1 (0.5%)	2 (0.6%)	1 (0.4%)	0 (0.0%)	3 (0.6%)	0 (0.0%)	1 (0.2%)	2 (0.9%)	2 (0.4%)	1 (0.6%)	3 (0.8%)	0 (0.0%)
**Census tract characteristics 5-year estimate (2015–2019)** **American Community Survey (ACS) data** ^**m**^														
**Composition by Racialized Group: Mean (SD)**														
Proportion White Non-Hispanic: Mean (SD)	0.59 (0.25)	0.65 (0.21)	0.46 (0.29)	0.62 (0.24)	0.57 (0.27)	0.58 (0.24)	0.62 (0.23)	0.53 (0.29)	0.58 (0.26)	0.62 (0.23)	0.59 (0.24)	0.59 (0.30)	0.61 (0.24)	0.58 (0.27)
Proportion Black Non-Hispanic: Mean (SD)	0.16 (0.21)	0.10 (0.12)	0.29 (0.29)	0.13 (0.18)	0.19 (0.25)	0.16 (0.19)	0.13 (0.16)	0.23 (0.27)	0.17 (0.23)	0.13 (0.17)	0.15 (0.20)	0.18 (0.25)	0.14 (0.18)	0.18 (0.24)
Proportion Hispanic: Mean (SD)	0.13 (0.11)	0.12 (0.12)	0.15 (0.11)	0.12 (0.11)	0.13 (0.11)	0.15 (0.13)	0.13 (0.12)	0.13 (0.10)	0.13 (0.11)	0.13 (0.12)	0.13 (0.11)	0.13 (0.12)	0.13 (0.11)	0.13 (0.12)
Proportion Asian: Mean (SD)	0.10 (0.09)	0.10 (0.09)	0.08 (0.08)	0.10 (0.09)	0.09 (0.08)	0.09 (0.09)	0.10 (0.09)	0.09 (0.08)	0.10 (0.09)	0.10 (0.09)	0.10 (0.09)	0.08 (0.08)	0.10 (0.09)	0.09 (0.09)
[Missing: N (%)]	[13 (1.9%)]	[8 ( 1.7%)]	[5 (2.3%)]	[4 (1.3%)]	[7 (2.8%)]	[2 (1.5%)]	[8 (1.7%)]	[5 (2.3%)]	[9 (1.9%)]	[4 (1.8%)]	[8 (1.5%)]	[5 (2.8%)]	[4 (1.0%)]	[3 (1.2%)]
**Index for Concentration at the Extremes (ICE): Mean (SD)**														
ICE for Racialized Economic Segregation (High-Income White Non-Hispanic vs. Low-Income Black Non-Hispanic) ^n^	0.24 (0.25)	0.29 (0.22)	0.13 (0.28)	0.26 (0.25)	0.22 (0.27)	0.23 (0.23)	0.26 (0.23)	0.19 (0.29)	0.23 (0.26)	0.26 (0.22)	0.24 (0.24)	0.23 (0.28)	0.26 (0.25)	0.22 (0.26)
ICE for Housing Tenure (Home Ownership vs. Renter)	-0.05 (0.49)	-0.03 (0.49)	-0.07 (0.47)	-0.02 (0.51)	-0.05 (0.47)	-0.08 (0.46)	-0.04 (0.48)	-0.06 (0.50)	-0.05 (0.49)	-0.05 (0.47)	-0.08 (0.48)	0.05 (0.50)	-0.05 (0.49)	-0.04 (0.46)
[Missing: N (%)]	[13 (1.9%)]	[8 ( 1.7%)]	[5 (2.3%)]	[4 (1.3%)]	[7 (2.8%)]	[2 (1.5%)]	[8 (1.7%)]	[5 (2.3%)]	[9 (1.9%)]	[4 (1.8%)]	[8 (1.5%)]	[5 (2.8%)]	[4 (1.0%)]	[3 (1.2%)]
**Median Income (2019 Inflation Adjusted US Dollars): Mean (SD)**	43 544.3 (15 581.6)	39 363.1 (14 377.5)	45 465.9 (15 50.8)	44 711.3 (15 294.8)	42 872.5 (15,945.8)	42 046.8 (15 472.8)	44 071.5 (14 758.9)	42 374.5 (17 293.1)	43 789.90 (15 881.48)	43 017.1 (14 939.4)	43 534.0 (15 474.1)	43 574.7 (15 938.8)	44 668.5 (16 052.3)	41 942.6 (14 597.0)
[Missing: N (%)]	[13 (1.9%)]	[8 ( 1.7%)]	[5 (2.3%)]	[4 (1.3%)]	[7 (2.8%)]	[2 (1.5%)]	[8 (1.7%)]	[5 (2.3%)]	[9 (1.9%)]	[4 (1.8%)]	[8 (1.5%)]	[5 (2.8%)]	[4 (1.0%)]	[3 (1.2%)]

^a^ The heterosexism analysis compares experiences of discrimination based on sexual orientation. The contrasted social comparison groups for this type of discrimination include individuals that identified as Straight or LGBQ. The Straight” category includes participants that only identified as straight or heterosexual, and the “LGBQ” category includes participants that identified as gay or lesbian, bisexual, queer, same gender loving, asexual, pansexual, questioning, or two or more sexualities. *N* = 2 participants did not report their sexuality and were not included in the comparison for heterosexism

^b^ The cissexism analysis compares experiences of discrimination based on gender modality. The contrasted social comparison groups for this type of discrimination include individuals that identify as cisgender and those that do not (transgender, nonbinary, or genderqueer individuals)

^c^ The sizeism analysis compares experiences of discrimination based on weight. The contrasted social comparison groups for this type of discrimination include those labeled as “obese” and “not obese” based on BMI (body mass index) ≥ 30 or < 30. *N* = 52 participants were missing height or weight measurements needed to calculate BMI, so these participants were not included in the comparison for sizeism

^d^ The Age, BMI, & Social Group Membership section reports observed values for persons included in the specified comparison groups and only reports missing data for body mass index (BMI), as this was the only variable in which there was missing data

^e−n^ See Appendix D Table 3

### The distribution of implicit and explicit measures and comparisons across social groups

**Fig. 1:**
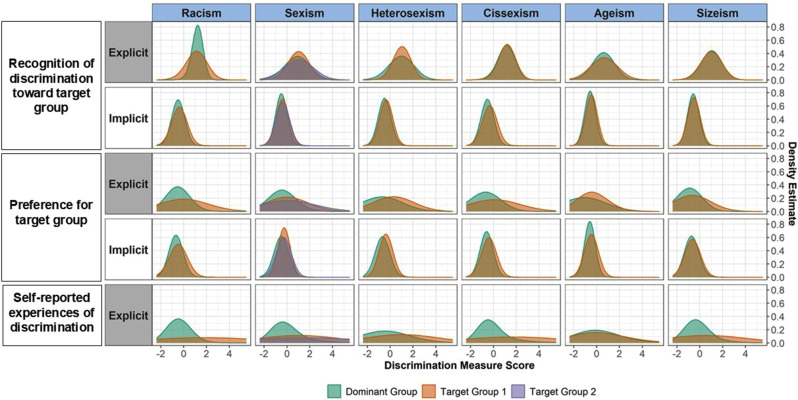
Standardized density plots displaying the distribution of implicit (B-IAT) and explicit exposures stratified by target vs. dominant groups for 6 types of discrimination in Life + Health Study participants (US-Born ages 25–64 years recruited from 3 community health centers), Boston, Massachusetts, 2020–2022

The distribution of implicit and explicit discrimination measures by experiment (discrimination type) and across the a priori selected social groups are displayed visually using standardized density plots in Fig. 1. Differences in implicit and explicit discrimination measures by a priori selected social groups are displayed in Appendix B. For the racism, sexism, heterosexism, cissexism, and ageism experiments, the target groups (e.g., people of color, women and nonbinary/genderqueer individuals, LGBQ individuals, individuals who were not cisgender, and those ages 45–64 years) had a significantly higher implicit preference for the target group and implicit recognition of members of the non-dominant social group as the targets of discrimination (denoted by positive scores on both). For the sizeism experiment, those “obese” vs. “not obese” (BMI > = 30 vs. BMI < 30) had a significantly greater implicit recognition for “obese” individuals (positive score) but lower implicit preference of “obese” individuals as the targets of discrimination (negative scores). Target groups generally reported more explicit experiences of discrimination than dominant groups, and self-reported preference for the dominant group.

### Correlations of implicit and explicit discrimination measures across the six experiments and stratified by group membership (Target and dominant group) and by explicit experiences of discrimination (EOD = 0 and EOD > = 1)

**Fig. 2:**
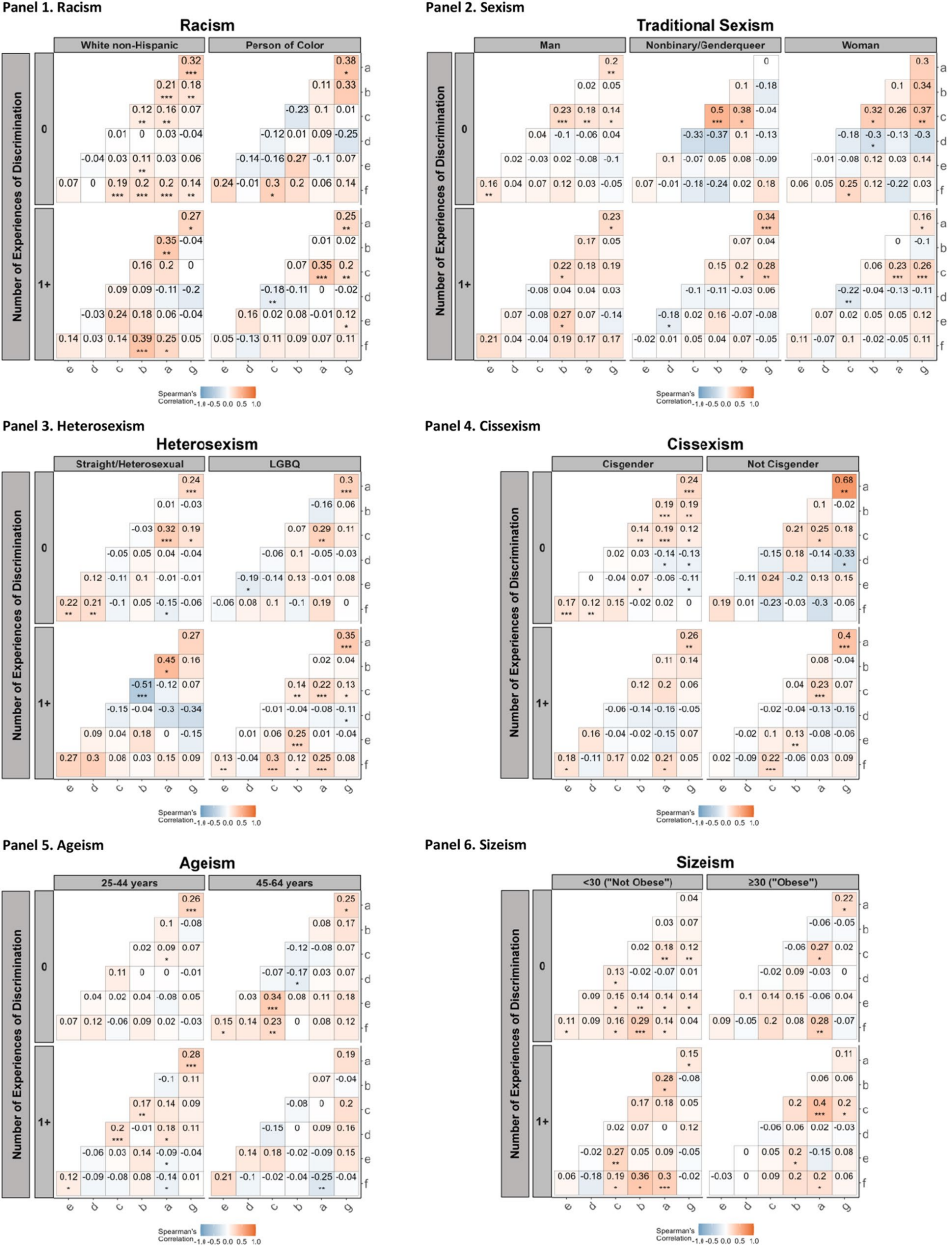
Correlations of implicit and explicit discrimination metrics, social desirability, sociopolitical concerns, and number of target group memberships by type of discrimination, stratified by target vs. dominant group discrimination and explicit discrimination, in Life + Health Study participants (US-Born ages 25–64 years recruited from 3 community health centers), Boston, Massachusetts, 2020–2022. **Axis Legend**: a = Implicit preference for target group; b = Explicit recognition of discrimination towards target group; c = Explicit preference for target group; d = Sociopolitical Concern; e = Social Desirability; f = N of group membership in target groups; g = Implicit recognition of discrimination towards target group

Figure 2 presents correlation matrices of implicit and explicit discrimination measures, sociopolitical concerns, social desirability bias, and number of target group memberships. Matrices are presented for each discrimination type (racism, sexism, heterosexism, cissexism, ageism, and sizeism) and are stratified by group membership (target and dominant group) and by explicit discrimination (EOD = 0 and EOD > = 1). Across experiments, there were generally modest correlations between discrimination measures. These correlations typically were stronger among those persons who self-reported no exposure to discrimination (EOD = 0) than for those who did (EOD > = 1). Results varied by membership in the target vs. dominant group. Social desirability bias only exhibited correlations for the target groups for traditional sexism and cissexism. For sociopolitical concerns, correlations were generally found with explicit discrimination.

### Modeling the associations of implicit and explicit discrimination with current smoking/vaping

**Fig. 3:**
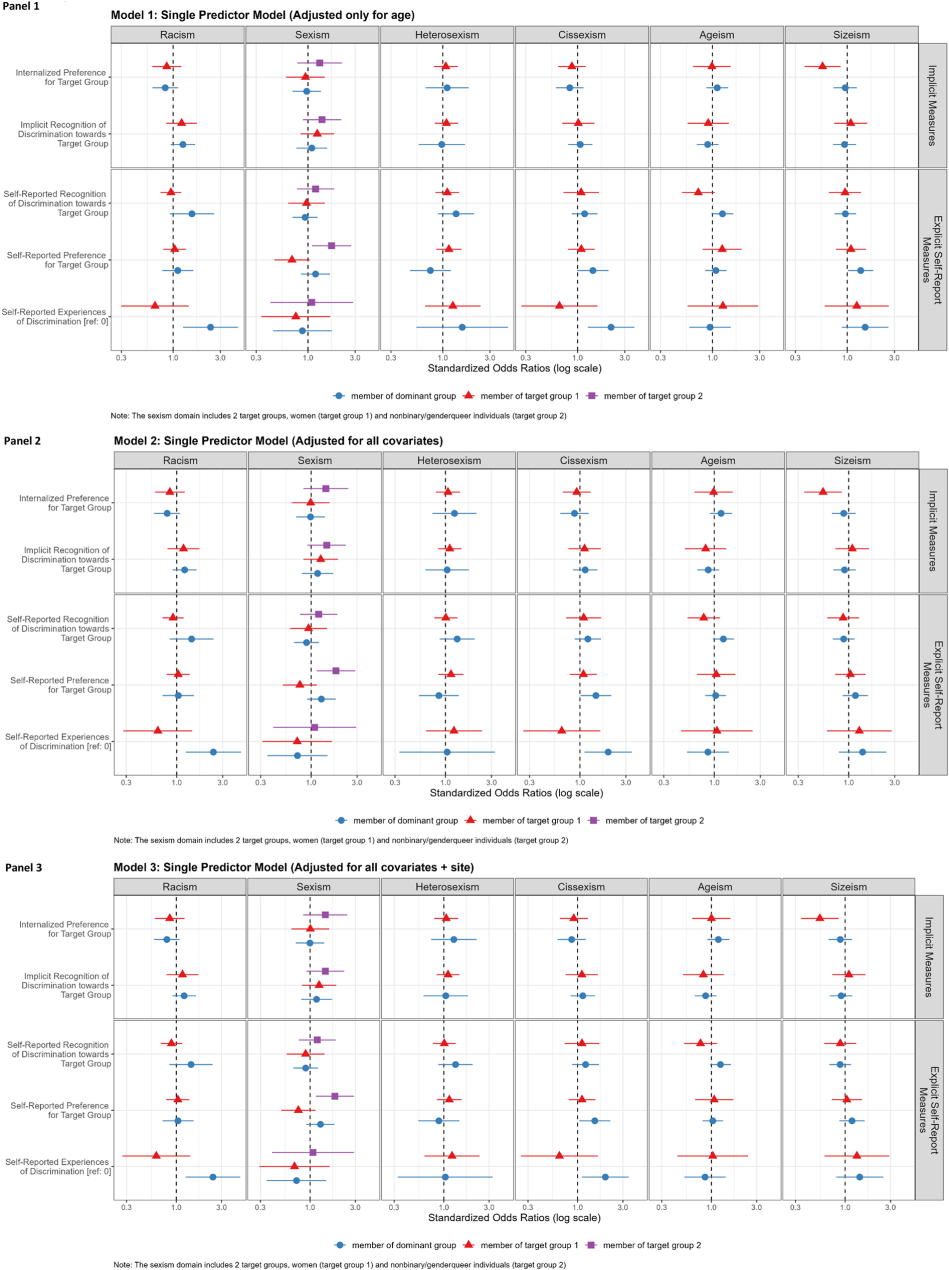

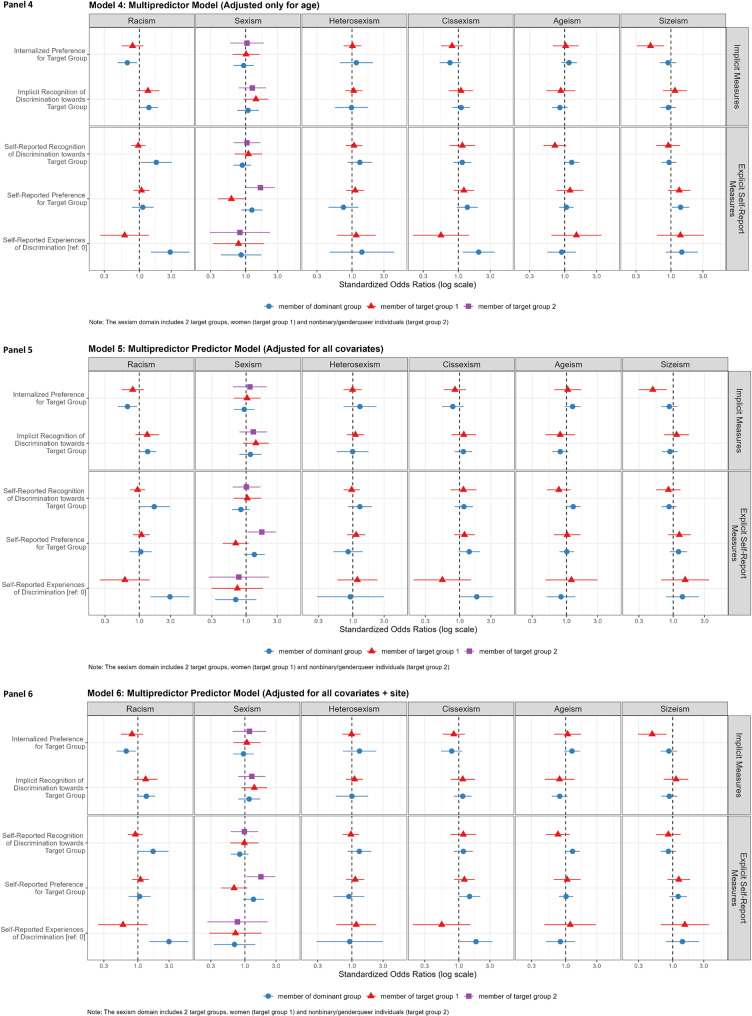
Associations of implicit (B-IAT) and explicit exposures with odds of smoking/vaping stratified by target vs. dominant group discrimination and explicit discrimination, in Life + Health Study participants (US-Born ages 25–64 years recruited from 3 community health centers), Boston, Massachusetts, 2020–2022

Overall, 15.4% of the study population (*n* = 108) reported current cigarette/vaping: 8.7% current smoking (4.1% every day, 4.6% some days), and 8.7% vaping (2.7% every day, 6.0% some days). Smoking/vaping prevalence was highest in people of color and nonbinary/genderqueer, LGBQ, and non-cisgender people. Figure 2,3 and Appendix C presents models assessing the association of implicit and explicit measures with current smoking/vaping and effects across target vs. dominant groups. The associations of discrimination exposures with smoking/vaping varied for implicit and explicit discrimination measures, were heterogeneous across experiments, and differed for target vs. dominant groups.

### Random-effects meta-regression analysis

We conducted a meta-analysis to analyze results generated from Model 6 (adjusted for age, education, number of target group memberships, sociopolitical concerns, social desirability, and recruitment site) across all six types of discrimination while preserving the heterogeneity of results across experiments to look at the overall pooled effect of implicit and explicit measures with current smoking/vaping, for target and dominant groups (see Table 2 and Appendix D). The point estimates of the target group effects were equivalent in value for the implicit and explicit measures. However, for the dominant group, the implicit and explicit measures yielded different odds of smoking/vaping–elevated odds for explicit discrimination measures, and null associations for the implicit measures. Additionally, we conducted a meta-regression to understand by how much the odds of smoking/vaping change when using explicit vs. implicit measures among target group and dominant group members, respectively. Among dominant group members, using explicit compared to implicit discrimination measures was associated with a 1.18 (95% CI = 1.00-1.39) greater odds of smoking/vaping; among target group members, explicit vs. implicit measures did not yield significantly different odds of smoking/vaping 1.00 (95% CI = 0.84–1.26).

**Table 2:** Random-effects meta-regression analysis: pooled associations of implicit (B-IAT) and explicit exposures with odds of smoking/vaping in Life + Health study participants (US-Born ages 25–64 years recruited from 3 community health centers), Boston, Massachusetts, 2020–2022

Summary Models	Interaction Model		
Measure Type Effect	Beta Estimate (95% CI)+	Estimate (95% CI) ^	*p*-value
Effect of explicit vs. implicit among the dominant group	0.163 (0.004, 0.330)	1.18 (1.00, 1.39)	0.06
Effect of explicit vs. implicit among the target group	-0.003 (-0.18, 0.18)	1.00 (0.84, 1.20)	0.98

+Additive Scale: Estimates are on the log odds scale and represent the additive change in the log odds of current smoking/vaping

^Multiplicative Scale: Additive estimates are exponentiated and represent the change in the odds of current smoking/vaping

## Discussion

This population-based study in three Boston-area community health centers used the recently introduced B-IAT to enable us to assess the health impacts of implicit and explicit discrimination measures for 6 different types of discrimination: racism, sexism, heterosexism, cissexism, ageism, and sizeism. We found, as expected, that, first, implicit and explicit discrimination measures yielded distinct yet complementary insights about exposure to discrimination. Second, these measures were generally moderately associated with one another, with stronger associations for target group members and those reporting no explicit discrimination. Relatedly, social desirability bias also varied, especially for traditional sexism and cissexism. Third, there was substantial heterogeneity in how implicit and explicit measures were associated with smoking/vaping across different types of discrimination. This finding highlights that discrimination experiences are not monolithic, and it is important to examine different types of discrimination – and to use methods such as meta-regression to identify common patterns across multiple types of discrimination. Additionally, sociopolitical concerns were independently associated with smoking/vaping, underlining the need to consider the broader context in which discrimination occurs.

Our study found that the association of implicit and explicit discrimination exposure with smoking/vaping varied by target vs. dominant group memberships, a novel finding that underscores the importance of examining effect estimates stratified by membership in the target versus dominant group. Specifically, the association between explicit vs. implicit discrimination measures increased the odds of smoking/vaping among dominant group members, but not among target group members. Prior research has demonstrated associations between discrimination and more active or proactive coping strategies among minoritized communities [10, 64]. Thus, study participants in target groups may have utilized effective coping strategies to mitigate the stress of discrimination, buffering its health-harming effects. Future research is warranted to examine protective factors and health-promoting strategies that communities deploy to actively resist oppression in their lives.

Several limitations should be considered. First, the sample was restricted to Boston area community health center patients, limiting generalizability to other healthcare or geographic settings. However, consistent with community health centers nationwide [65], our sample from two of the three community health centers (Mattapan and Harvard Street) were primarily low-income persons of color, whereas our sample from Fenway comprised more highly educated white non-Hispanic LGBTQ + persons. Second, combining smoking and vaping as a single outcome variable may obfuscate important epidemiologic differences relevant to discrimination-health associations. Both smoking and vaping are concentrated in men, people identifying as non-Hispanic White and non-Hispanic “Other race” groups, those with low levels of education (e.g., high school or some college) and low income, and LGBT-identified people [27–30]. Yet, smoking prevalence is highest for individuals ages 25–44 and 45–46 years, whereas vaping is highest among those ages 18–24 years [27–30]. Additional research is warranted to examine the associations of implicit and explicit discrimination with smoking and vaping as disaggregated outcomes. Third, this is a cross-sectional study design and therefore associational only. Future longitudinal research is needed to assess the timing of implicit and explicit discrimination exposures and health, consider the mechanisms and pathways through which discrimination harms health over time, and examine temporal trends in the sociopolitical landscape that may be relevant for discrimination-health associations given our findings that sociopolitical concerns associated with some types of discrimination and with current smoking/vaping. Limitations notwithstanding, this study has noteworthy strengths, including the use of a novel brief IAT to measure implicit recognition alongside self-report explicit discrimination exposures, a diverse sample that allows for statistical comparisons across a priori selected social groups, and a novel methodology of using meta-regression to pool results across the six discrimination experiments to obtain pooled estimates of implicit and explicit discrimination measures, stratified by target vs. dominant groups, with current smoking/vaping.

## Conclusion

Our results affirm the need for empirical health research to include both implicit and explicit measures of exposure to discrimination and demonstrate the practical feasibility of doing this by using the B-IAT plus self-report measures. We provide novel evidence that it is critical to analyze results separately for members of the target vs. dominant groups, and that it is useful to make these comparisons across multiple types of discrimination using the novel meta-analysis approach we have employed. While prior studies have assessed implicit bias and demonstrated that these can vary based on group membership [66–69], our study is distinct by examining implicit beliefs about which social groups are implicitly recognized or believed to be the targets of discrimination. Further, our study provides suggestive evidence that it is important to include relevant contextual measures, e.g., about sociopolitical anxiety, in studying implicit and explicit discrimination measures and health, since such metrics can potentially be confounders or effect modifiers [70–73]. Lastly, these novel implicit and explicit measures enable future research investigations assessing the intersectional health effects of occupying multiple social positions in target vs. dominant groups, and the intersecting forms of societal oppression that may fuel health inequities, representing an important next step for the field. Future research on discrimination and health, in diverse country contexts, should consider using both implicit and explicit measures to analyze health impacts across multiple types of discrimination.

## Electronic supplementary material

Below is the link to the electronic supplementary material.

Supplementary Material 1

## Data Availability

No study data will be made available until study completion and will only be available if a data request is approved by the study team. For data-related queries, please contact the corresponding author.
