# Supplementary material for: Analyzing multiple types of discrimination using implicit and explicit measures, comparing target vs. Dominant groups, in a study of smoking/vaping among community health center members in Boston, Massachusetts (2020–2022)
Source: Int J Equity Health. 2025 Apr 22;24:110. doi: 10.1186/s12939-025-02456-9 (PMC12016388; doi:10.1186/s12939-025-02456-9)
Supplement: Supplementary file 1 — Supplementary Material 1 [file 12939_2025_2456_MOESM1_ESM.docx]

**APPENDIX**

| **APPENDIX A: TECHNICAL DESCRIPTION OF THE MEASURES EMPLOYED** |
| --- |

**Terminology**

In the U.S., the terminology used to describe discrimination and experiences of discrimination continues to evolve over time, across different sociopolitical contexts, and in various geographic regions. Depending on time and place, the kinds of discrimination involved can be legal (de jure), illegal (but still occurring, de facto) or not defined in legal terms (also de facto). In this study, we examined six types of discrimination which systemically privilege dominant social groups and are directed against targeted socially non-dominant groups. The terms we use and our definitions are as follows:

1. **Racism:** Discrimination on the basis of race, privileging white people over people of color; sometimes referred to as race-based discrimination.
2. **Sexism:** Discrimination on the basis of sex, privileging men over women and nonbinary/genderqueer people; sometimes referred to as sex- or gender-based discrimination.
3. **Cissexism:** Discrimination on the basis of gender modality, privileging cisgender people over those who are not cisgender; sometimes referred to as transphobia, gender binarism, or gender identity-related discrimination.
4. **Heterosexism:** Discrimination on the basis of sexual orientation, privileging straight/heterosexual people over people who are sexual minority (LGBQ+ people); sometimes referred to as homophobia or sexual orientation-based discrimination.
5. **Ageism:** Discrimination on the basis of age, privileging younger people over older people; sometimes referred to as age-based discrimination.
6. **Sizeism:** Discrimination on the basis of size or weight, privileging people who are not overweight/obese over people who are overweight/obese; sometimes referred to as fatphobia or weight-based discrimination.

**Implicit Discrimination Measures**

Implicit discrimination was measured using The Brief Implicit Association Test (B-IAT).1 The B-IAT is a shortened version of the standard IAT,2 designed to measure automatic mental representations and associations more efficiently while maintaining reliability and validity. The B-IAT typically takes about two minutes to complete, compared to the 15 minutes required for the standard IAT, as it includes fewer blocks and trials.1,3 Despite its shorter duration, the B-IAT uses the same basic methodology, where participants rapidly categorize stimuli (words or images) on the screen. The key difference is that participants focus on a specific set of target categories and attributes at a time. Separate B-IAT experiments assessed six social discrimination types: racism, sexism, heterosexism, cissexism, ageism, and sizeism. For each type, a Target/Dominant B-IAT assessed an implicit recognition of discrimination toward members of the target group, and a Good/Bad B-IAT measured internalized preference for the target group. B-IAT scores were computed only for participants with an error rate below 30% and with <10% of responses being too rapid and thus invalid; the percentage of scores missing for each B-IAT ranged from 4-9%. Scores could range from +2 to −2 with a 0 indicating neutrality between attributes and social categories. Positive scores indicated an implicit preference for the target group and an implicit recognition of members of the non-dominant social group as the targets of discrimination. Negative scores indicated an implicit preference for the dominant group and an implicit recognition of the dominant social group as the target of discrimination. Each brief IAT took approximately two minutes to administer, totaling 12 minutes.

**Explicit Self-Reported Discrimination and Preference Measures**

The validated Experiences of Discrimination (EOD) measure assessed explicit self-reported discrimination.4 Ten items assessed whether participants had ever “experienced discrimination, been prevented from doing something, or been hassled or made to feel inferior in any of the following situations” querying the frequency of occurrence across 10 domains due to their race, gender identity, sexuality, gender modality, age, and weight. The 10 domains were: (1) school; (2) getting hired or getting a job; (3) at work; (4) getting housing; (5) getting medical care; (6) getting service in a store or restaurant; (7) getting credit, bank loans, or a mortgage; (8) on the street or in a public setting’ (9) from the police or in the courts or getting or changing legal documents; and (10) at home. Explicit recognition of discrimination against targeted groups was measured using self-reported data. Participants were asked to report how often they felt that people belonging to the targeted non-dominant social groups experienced discrimination due to their race, gender identity, sexuality, gender modality, age, and weight. Responses were obtained on a 4-point scale and coded as scores from 0 to 3 (i.e., 0 “Never”, 1 “Rarely”, 2 “Sometimes”, and 3 “Often”). Explicit preferences were measured using self-reported items. Participants were asked to select which statement best described them from seven options ranging from strong preference for one social group to strong preference for another social group. Responses were coded as scores from −3 to +3 with more positive scores indicating stronger preferences for the non-dominant social group (e.g., in the racism experiment, more positive scores indicating stronger preferences for People of Color over White People).

**Social Groups**

**Target vs. dominant groups were operationalized corresponding the six categories of social discrimination.**

**Racism.** For racism comparisons, White non-Hispanic vs People of Color (inclusive of all racial ethnic groups other than White non-Hispanic) were compared. Participants were asked to report all racialized groups with which they self-identified, using the US census categories. Participants belonging to more than one racialized group (n=58) were categorized as “Two or More Racialized Groups.” No participants self-identified as Native Hawaiian or Pacific Islander, however n=4 participants self-identified as American Indian/Alaskan Native in addition to another racialized group. Participants that identified as “Two or More Racialized Groups” included the following: White and Hispanic: n=17; White and Asian: n=14; Hispanic and Black: n=10; White non-Hispanic and Black: n=9; White non-Hispanic and AIAN: n=2; Black non-Hispanic and AIAN: n=2; White non-Hispanic and MENA: n=1; Black non-Hispanic and Asian: n=1; Black non-Hispanic and mixed race: n=1; Asian and MENA: n=1.

**Sexism.** Gender identity was asked using the two-step method5,6 as cisgender man, cisgender woman, transgender woman, transgender man, nonbinary/transgender nonbinary/genderqueer. For sexism comparisons, woman (inclusive of transgender woman) or nonbinary/genderqueer individuals compared to a man (inclusive of transgender man).

**Cissexism.** For cissexism comparisons, the contrasted social comparison groups were individuals that identify as cisgender vs those that do not (transgender, nonbinary, or genderqueer individuals).

**Heterosexism.** For heterosexism comparisons, sexual orientation was operationalized as straight/heterosexual vs LGBQ (all other categories). Participants were asked to report all sexual identity groups with which they self-identified. Participants identifying with two or more sexualities (n=97) were categorized as “Two or more sexualities.” Participants that identified as “Two or More Sexualities” includes the following: Queer and gay/lesbian: n=30; Bisexual and queer: n=28; Queer, same gender loving, gay/lesbian: n=8; Queer and straight: n=4; Bisexual, queer, same gender loving, and gay/lesbian: n=3; Bisexual, queer, gay/lesbian: n=3; Same gender loving and gay/lesbian: n=3; Bisexual and gay/lesbian: n=2; Queer and same gender loving: n=2; Queer and pansexual: n=2.

**Ageism.** Age was self-reported continuously in years and coded as ages 25-44 years vs ages 45-64 years for ageism comparisons.

**Sizeism.** For sizeism comparisons, we computed body mass index (BMI; weight/height2) based on self-reported height and weight data, and dichotomized <30 BMI (“not obese” who were underweight, healthy weight, overweight) vs. >=30 BMI (“obese”).7 Participants missing height or weight measurements needed to calculate BMI (n=52) were not included in the comparison for sizeism. We also created a variable summing the number of target groups a person reported being in from 0-6.

**Sociopolitical Concerns**

We assessed sociopolitical concerns using a 15-item scale from the Gallup Poll Social Series,8 fielded since 2001, about concerns with problems facing the country.9 Participants were asked how much they personally worry about each problem with response options “not at all”, “only a little”, “a fair amount”, or “a great deal”. Problems listed included topics related to hunger and homelessness, the environment, immigration, race relations, healthcare, and unemployment. Responses were coded from 0=not at all, 1=only a little, 2=a fair amount, 3=a great deal and summed to create a political concern score ranging from 0-45 with higher scores indicating greater concern. Internal consistency reliability of the measure in this sample was high (α=0.87).

**Social Desirability Bias**

We used the validated RAND Socially Desirable Response Set Five-Item Survey (SDRS-5) to measure social desirability bias.10 Participants were asked how true each statement was to them (e.g., “I am always courteous even to people who are disagreeable”). Responses were “Definitely True”, “Mostly True”, “Don’t Know”, “Mostly False” and “Definitely False”. Participants that responded “Definitely True” to items 1 and 5 and responded “Definitely False” to items 2,3, and 4 were given an item score of 1, all other responses were coded as 0. Items were summed (0-5) and transformed to a log linear scale (0-100).

**Sociodemographic Characteristics and Context**

Participants self-reported individual educational attainment (graduate degree, four years of college, some college/vocational school, no college)11 and current relationship status (married, in a relationship, single, divorced/separated, widowed, other).4 For socioeconomic measures, participants were asked four items about their childhood (up to age 11 years) and adult economic situation.11 If any of the following situations happened at least twice during childhood or adulthood, respectively, participants were considered to have had childhood or adult economic deprivation, respectively: (1) We did not have enough money to pay for food, or for rent or mortgage; (2) We had to borrow money to pay for medical expenses; (3) We did not have enough money to make ends meet; (4) We received public assistance or welfare. Participants were asked three items about their past 12-month food situation at the time of the survey.12 Participants that responded “Often” or “Sometimes to any of the following questions were considered food insecure: In the past 12 months (1) I worried whether my food would run out before I got money to buy more; (2) The food that I bought just didn’t last and I didn’t have enough money to get more food; (3) I couldn’t afford to eat balanced meals. Occupational class and housing tenure were assessed using questions from the Office of National Statistics11,13 and the US Census Bureau, respectively.14 To characterize participants’ residential context, we geocoded the mailing address they provided at recruitment to the U.S. Census tract, and utilized the 5-year estimate (2015-2019) American Community Survey (ACS) data for: composition by racialized group, Index for Concentration at the Extremes (ICE) for racialized economic segregation and housing tenure,15 and median income (2019 inflation-adjusted US dollars). For the ICE for racialized economic segregation, high-income refers to the top quintile for US household income and low-income refers to the bottom quintile for US household income, during the years specified; for further explication, see: https://www.hsph.harvard.edu/thegeocodingproject/covid-19-resources/ (accessed January 17, 202). n=13 participants were missing census tract identifiers, so we were unable to compute census tract metrics for these participants.

**Health Behavior: Current Smoking/Vaping**

Four items drawn from the U.S. Behavioral Risk Factor Surveillance Survey asked about lifetime and current cigarette use (smoking) and e-cigarette (vaping).16 Smoking was asked as, “Have you smoked at least 100 cigarettes in your entire life?” and “Do you now smoke cigarettes every day, some days, or not at all?” Participants were asked about vaping, “Have you ever used an e-cigarette or other electronic vaping product, even just one time, in your entire life?” and “Do you now use e-cigarettes or other electronic vaping products every day, some days, or not at all?” Variables were combined to create a binary indicator of current smoking/vaping to capture a current stress-responsive health behavior. Participants who reported smoking cigarettes or vaping “every day” or “some days” (regardless of the number of lifetime cigarettes smoked) were coded as endorsing current smoking/vaping and compared to those who reported never use or “not at all” current use.

**References for Measures**

1. Marini M, Waterman PD, Breedlove E, et al. The target/perpetrator brief-implicit association test (B-IAT): an implicit instrument for efficiently measuring discrimination based on race/ethnicity, sex, gender identity, sexual orientation, weight, and age. BMC Public Health. Jan 19 2021;21(1):158. doi:10.1186/s12889-021-10171-7

2. Greenwald AG, McGhee DE, Schwartz JL. Measuring individual differences in implicit cognition: the implicit association test. J Pers Soc Psychol. Jun 1998;74(6):1464-80. doi:10.1037//0022-3514.74.6.1464

3. Marini M, Waterman PD, Breedlove ER, et al. Using Implicit Measures of Discrimination: White, Black, and Hispanic Participants Respond Differently to Group-Specific Racial/Ethnic Categories vs. the General Category "People of Color" in the USA. J Racial Ethn Health Disparities. Aug 2023;10(4):1682-1692. doi:10.1007/s40615-022-01353-z

4. Krieger N, Smith K, Naishadham D, Hartman C, Barbeau EM. Experiences of discrimination: validity and reliability of a self-report measure for population health research on racism and health. Soc Sci Med. Oct 2005;61(7):1576-96. doi:10.1016/j.socscimed.2005.03.006

5. National Academies of Sciences E, and Medicine (NASEM),. Measuring Sex, Gender Identity, and Sexual Orientation. National Academies of Sciences, Engineering, and Medicine; Division of Behavioral and Social Sciences and Education; Committee on National Statistics; Committee on Measuring Sex, Gender Identity, and Sexual Orientation; 2022.

6. Reisner SL, Conron KJ, Tardiff LA, Jarvi S, Gordon AR, Austin SB. Monitoring the health of transgender and other gender minority populations: validity of natal sex and gender identity survey items in a U.S. national cohort of young adults. BMC Public Health. Nov 26 2014;14:1224. doi:10.1186/1471-2458-14-1224

7. Centers for Disease Control and Prevention. Defining Adult Overweight & Obesity. CDC. Accessed May 10, 2024. https://www.cdc.gov/obesity/basics/adult-defining.html

8. Gallup. How does the Gallup Poll Social Series work? . 2023. Accessed July 15, 2024. https://www.gallup.com/175307/gallup-poll-social-series-methodology.aspx

9. Gallup Organization. Gallup Poll Social Series: Environment. 2018. January 31, 2024. https://www.protectourcare.org/wp-content/uploads/2018/05/180326Worry.pdf

10. Hayes RD, Hayashi T, Stewart AL. A Five-Item Measure of Socially Desirable Response Set. Educational and Psychological Measurement. 1989;49(3):629-636.

11. Krieger N, Chen JT. Sociodemographic and cultural factors questionnaire, for the Breast Cancer Comprehensive Questionnaire. Prepared for the National Action Plan on Breast Cancer of the Office on Women’s Health, U.S. Department of Health and Human Services. 1996.

12. Centers for Disease Control and Prevention NCfHS. National Health and Nutrition Examination Survey Questionnaire (Examination Components and Laboratory Components, 2017-2018, "Food Insecurity - FSQ," FSQ.032. 2017.

13. Office for National Statistics United Kingdom. The National Statistics Socio-economic classification (NS-SEC) (Section 14; self-report method). 2019. August 5, 2019. https://www.ons.gov.uk/methodology/classificationsandstandards/otherclassifications/thenationalstatisticssocioeconomicclassificationnssecrebasedonsoc2010#deriving-the-ns-sec-self-coded-method

14. U.S. Census Bureau. Housing Characteristics: 2000. 2001. June 24, 2024. https://www2.census.gov/library/publications/decennial/2000/briefs/c2kbr01-13.pdf

15. Krieger N, Chen JT, Waterman PD. Using the methods of the Public Health Disparities Geocoding Project to monitor COVID-19 inequities and guide action for social justice. Accessed May 10, 2024. https://www.hsph.harvard.edu/thegeocodingproject/covid-19-resources/

16. Centers for Disease Control and Prevention. Behavioral Risk Factor Surveillance System Survey Questionnaire, 2019. 2019.

| **Appendix B: Supplemental Table 1 implicit and self-reported explicit measures of discrimination, for 6 types of discrimination, stratified by target vs. dominants groups, among Life + Health Study participants (US-Born Ages 25-64 Years), from three community health centers), Boston, Massachusetts, 2020-2022.** | | | | | | | | | | |
| --- | --- | --- | --- | --- | --- | --- | --- | --- | --- | --- |
|  |  | **Implicit Measures ^a^** | | | | **Self-Report Explicit Measures** | | | | |
|  |  | **Recognition of**  **Discrimination Against Members of Target Group** | | **Internalized Preference for Target Group** | | **Individual Self-Reported Experiences of Discrimination:**  **Number of Domains: N (%) ^b^** | | | **Recognition of Discrimination Against Target Group: Self-Reported Assessment of Frequency ^c^** | **Self-Reported Preference for Dominant Group ^d^** |
|  | **Total N** | **N** | **Mean (SD)** | **N** | **Mean (SD)** | **0** | **1-2** | **3+** | **Mean (SD)** | **Mean (SD)** |
| **Racism** |  | | | | | | | | | |
| White Non-Hispanic (ref) | 478 | 466 | 0.11 (0.41)  ** | 467 | -0.19 (0.45)  ** | 411  (86.0%)  †† | 56  (11.7%) | 11  (2.3%)  †† | 2.88  (0.36) | 0.09  (0.78) |
| Person of Color | 221 | 199 | 0.29 (0.42)  **†† | 197 | 0.13 (0.50)  **†† | 43  (19.5%) | 48  (21.7%) | 130 (58.8%) | 2.74  (0.58)  †† | 0.93  (1.34)  †† |
| **Sexism** |  | | | | | | | | | |
| Man (ref) | 313 | 293 | 0.12 (0.36)  ** | 296 | 0.11 (0.42)  ** | 208  (66.5%)  †† | 57  (18.2%)  †† | 48 (15.3%)  †† | 2.40  (0.76) | 0.20  (1.25) |
| Woman | 254 | 245 | 0.28 (0.36) **†† | 246 | 0.50 (0.36)  **†† | 59  (23.2%) | 63 (24.8%) | 132 (52.0%) | 2.57  (0.60)  † | 0.94  (1.18)  †† |
| Nonbinary/Genderqueer | 132 | 123 | 0.31 (0.37) **†† | 126 | 0.46 (0.38)  **†† | 36  (27.3%) | 21 (15.9%) | 75 (56.8%) | 2.55  (0.82) | 0.98  (1.39)  †† |
| **Heterosexism** |  | | | | | | | | | |
| Straight/Heterosexual (ref) | 219 | 194 | 0.11 (0.41) ** | 198 | -0.18 (0.40)  ** | 194 (88.6%)  †† | 13  (5.9%)  †† | 12 (5.5%)  †† | 2.51  (0.69) | -0.29  (1.14) |
| LGBQ | 478 | 458 | 0.34 (0.41)  ** †† | 463 | 0.30 (0.43)  **†† | 95  (19.9%) | 147 (30.8%) | 236 (49.4%) | 2.59  (0.57) | 1.39  (1.27)  †† |
| **Cissexism** |  | | | | | | | | | |
| Cisgender (ref) | 477 | 434 | 0.06 (0.43)  ** | 442 | -0.06 (0.41)  ** | 351 (73.6%)  †† | 56 (11.7%) | 70 (14.7%)  †† | 2.75  (0.56) | -0.26  (0.99) |
| Not Cisgender | 222 | 206 | 0.38 (0.41) **†† | 209 | 0.26 (0.47)  **†† | 32  (14.4%) | 48 (21.6%) | 142 (64.0%) | 2.86  (0.48)  † | 0.99  (1.41)  †† |
| **Ageism** |  | | | | | | | | | |
| 25-44 Years (ref) | 520 | 488 | -0.02 (0.35) | 498 | -0.06 (0.34)  ** | 296 (56.9%)  †† | 140 (26.9%)  †† | 84 (16.2%)  †† | 1.91  (0.70) | -0.79  (1.37) |
| 45-64 Years | 179 | 163 | 0.17 (0.33) **†† | 166 | 0.16 (0.37)  **†† | 102 (57.0%) | 48 (26.8%) | 29 (16.2%) | 1.99  (0.71) | 0.33  (1.28)  †† |
| **Sizeism** |  | | | | | | | | | |
| <30 ("Not Obese") (ref) | 400 | 385 | -0.03  (0.35) | 385 | -0.26 (0.44)  ** | 275 (68.8%)  †† | 88 (22.0%) | 37  (9.2%)  †† | 2.58  (0.63) | -0.62  (0.96) |
| ≥30 ("Obese") | 247 | 228 | 0.06 (0.34) *†† | 232 | -0.13 (0.44)  **†† | 97  (39.3%) | 64 (25.9%) | 86 (34.8%) | 2.56  (0.59) | -0.17  (1.04)  †† |
| **^a^** ^-d^ See Appendix D Table1-2 | | | | | | | | | | |

| **Appendix C: Supplemental Table 2. Bivariate and multivariate model estimates assessing the association of implicit and explicit discrimination measures with current smoking/vaping by target vs dominant group status in Life + Health Study participants (US-Born ages 25-64 years recruited from 3 community health centers), Boston, Massachusetts, 2020-2022.** | | | | | | | | | | | | | | |
| --- | --- | --- | --- | --- | --- | --- | --- | --- | --- | --- | --- | --- | --- | --- |
| **Domain, Discrimination Measure, and Social Group** | | | **Model 1: Single Predictor (adjusted only for age)** | | **Model 2: Single Predictor (adjusted for age, education, N of group membership in target groups, political anxiety & social desirability)** | | **Model 2.5: Single Predictor (adjusted for age, education, N of group membership in target groups, political anxiety, social desirability & recruitment site** | | **Model 3: Multi-predictor (adjusted only for age)** | | **Model 4: Multi-predictor (adjusted for age, education, N of group membership in target groups, political anxiety & social desirability)** | | **Model 4.5: Multi-predictor (adjusted for age, education, N of group membership in target groups, political anxiety, social desirability & recruitment site)** | |
| ***Domain*** | ***Discrimination Measure*** | ***Social Group*** | ***Estimate [95% CI]*** | ***P*** | ***Estimate [95% CI]*** | ***P*** | ***Estimate [95% CI]*** | ***P*** | ***Estimate [95% CI]*** | ***P*** | ***Estimate [95% CI]*** | ***P*** | ***Estimate [95% CI]*** | ***P*** |
| Racism |  | | | | | | | | | | | | | |
|  | Implicit Recognition of Racism toward POC | White non-Hispanic | 1.26 [0.95,1.67] | 0.11 | 1.21 [0.91,1.61] | 0.19 | 1.21 [0.91,1.61] | 0.19 | 1.39 [1.02,1.89] | 0.04 | 1.34 [0.99,1.83] | 0.06 | 1.35 [0.99,1.83] | 0.06 |
|  | Implicit Recognition of Racism toward POC | Person of Color | 1.22 [0.86,1.73] | 0.27 | 1.18 [0.8,1.72] | 0.4 | 1.16 [0.79,1.71] | 0.44 | 1.35 [0.91,1.98] | 0.13 | 1.32 [0.87,2] | 0.18 | 1.31 [0.86,1.99] | 0.2 |
|  | Internalized Preference for POC | White non-Hispanic | 0.83 [0.62,1.12] | 0.23 | 0.8 [0.58,1.08] | 0.15 | 0.8 [0.58,1.09] | 0.15 | 0.66 [0.47,0.93] | 0.02 | 0.66 [0.47,0.93] | 0.02 | 0.66 [0.47,0.93] | 0.02 |
|  | Internalized Preference for POC | Person of Color | 0.86 [0.62,1.21] | 0.39 | 0.85 [0.59,1.21] | 0.37 | 0.85 [0.6,1.22] | 0.39 | 0.79 [0.55,1.15] | 0.22 | 0.8 [0.54,1.17] | 0.25 | 0.81 [0.55,1.2] | 0.29 |
|  | Self-Reported Recognition of Racism toward POC | White non-Hispanic | 1.55 [0.93,2.59] | 0.1 | 1.43 [0.85,2.41] | 0.18 | 1.43 [0.85,2.41] | 0.18 | 1.79 [1.05,3.05] | 0.03 | 1.7 [0.98,2.93] | 0.06 | 1.71 [0.99,2.95] | 0.06 |
|  | Self-Reported Recognition of Racism toward POC | Person of Color | 0.95 [0.75,1.21] | 0.68 | 0.92 [0.71,1.18] | 0.5 | 0.89 [0.69,1.15] | 0.38 | 0.96 [0.75,1.24] | 0.77 | 0.94 [0.73,1.23] | 0.66 | 0.91 [0.7,1.19] | 0.49 |
|  | Self-Reported Preference for POC | White non-Hispanic | 1.11 [0.78,1.6] | 0.55 | 1.04 [0.71,1.51] | 0.84 | 1.04 [0.72,1.52] | 0.83 | 1.13 [0.78,1.64] | 0.53 | 1.06 [0.72,1.55] | 0.78 | 1.06 [0.72,1.56] | 0.77 |
|  | Self-Reported Preference for POC | Person of Color | 1.04 [0.8,1.35] | 0.79 | 1.04 [0.79,1.36] | 0.8 | 1.04 [0.79,1.38] | 0.78 | 1.08 [0.81,1.43] | 0.6 | 1.08 [0.8,1.45] | 0.61 | 1.09 [0.81,1.47] | 0.57 |
|  | Self-Reported Experiences of Racism [ref: 0] | White non-Hispanic | 2.39 [1.26,4.53] | 0.01 | 2.4 [1.24,4.65] | 0.01 | 2.43 [1.26,4.71] | 0.01 | 2.89 [1.49,5.64] | 0 | 2.94 [1.48,5.83] | 0 | 2.99 [1.5,5.93] | 0 |
|  | Self-Reported Experiences of Racism [ref: 0] | Person of Color | 0.66 [0.3,1.44] | 0.29 | 0.64 [0.28,1.45] | 0.28 | 0.62 [0.27,1.4] | 0.25 | 0.6 [0.26,1.39] | 0.23 | 0.6 [0.25,1.44] | 0.25 | 0.59 [0.25,1.41] | 0.24 |
| Sexism |  | | | | | | | | | | | | | |
|  | Implicit Recognition of Sexism toward Women | Man | 1.09 [0.76,1.57] | 0.62 | 1.17 [0.8,1.69] | 0.41 | 1.17 [0.81,1.7] | 0.4 | 1.09 [0.75,1.58] | 0.66 | 1.17 [0.79,1.72] | 0.44 | 1.16 [0.79,1.72] | 0.45 |
|  | Implicit Recognition of Sexism toward Women | Nonbinary/  genderqueer | 1.24 [0.84,1.84] | 0.28 | 1.26 [0.83,1.9] | 0.27 | 1.25 [0.82,1.89] | 0.3 | 1.42 [0.93,2.18] | 0.1 | 1.41 [0.9,2.21] | 0.13 | 1.39 [0.89,2.19] | 0.15 |
|  | Implicit Recognition of Sexism toward Women | Woman | 1.39 [0.89,2.17] | 0.15 | 1.45 [0.92,2.3] | 0.11 | 1.45 [0.92,2.29] | 0.11 | 1.26 [0.78,2.01] | 0.34 | 1.29 [0.8,2.1] | 0.3 | 1.28 [0.79,2.07] | 0.32 |
|  | Internalized Preference for Women | Man | 0.97 [0.7,1.35] | 0.87 | 0.99 [0.7,1.39] | 0.94 | 1 [0.71,1.41] | 0.99 | 0.93 [0.66,1.33] | 0.71 | 0.94 [0.65,1.35] | 0.74 | 0.95 [0.66,1.37] | 0.79 |
|  | Internalized Preference for Women | Nonbinary/  genderqueer | 0.94 [0.6,1.48] | 0.8 | 0.99 [0.63,1.55] | 0.96 | 1.01 [0.64,1.59] | 0.97 | 1.02 [0.64,1.62] | 0.95 | 1.04 [0.64,1.67] | 0.89 | 1.07 [0.66,1.74] | 0.78 |
|  | Internalized Preference for Women | Woman | 1.31 [0.78,2.21] | 0.3 | 1.42 [0.83,2.43] | 0.2 | 1.45 [0.85,2.47] | 0.17 | 1.05 [0.59,1.87] | 0.88 | 1.15 [0.63,2.07] | 0.65 | 1.17 [0.65,2.12] | 0.59 |
|  | Self-Reported Recognition of Sexism toward Women | Man | 0.94 [0.7,1.25] | 0.66 | 0.9 [0.67,1.21] | 0.47 | 0.9 [0.67,1.21] | 0.49 | 0.89 [0.66,1.21] | 0.47 | 0.83 [0.61,1.14] | 0.25 | 0.83 [0.61,1.14] | 0.25 |
|  | Self-Reported Recognition of Sexism toward Women | Nonbinary/  genderqueer | 0.97 [0.63,1.48] | 0.88 | 0.94 [0.6,1.46] | 0.78 | 0.9 [0.57,1.42] | 0.65 | 1.1 [0.69,1.76] | 0.7 | 1.04 [0.64,1.7] | 0.86 | 0.98 [0.59,1.62] | 0.94 |
|  | Self-Reported Recognition of Sexism toward Women | Woman | 1.2 [0.78,1.84] | 0.42 | 1.2 [0.77,1.88] | 0.43 | 1.19 [0.76,1.86] | 0.44 | 1.04 [0.66,1.65] | 0.85 | 1.01 [0.62,1.65] | 0.96 | 0.98 [0.61,1.6] | 0.95 |
|  | Self-Reported Preference for Women | Man | 1.19 [0.85,1.67] | 0.3 | 1.28 [0.91,1.8] | 0.16 | 1.29 [0.91,1.81] | 0.15 | 1.24 [0.86,1.78] | 0.25 | 1.34 [0.93,1.93] | 0.12 | 1.35 [0.93,1.95] | 0.11 |
|  | Self-Reported Preference for Women | Woman | 0.69 [0.46,1.04] | 0.08 | 0.77 [0.51,1.15] | 0.2 | 0.75 [0.5,1.14] | 0.18 | 0.61 [0.39,0.97] | 0.04 | 0.7 [0.44,1.1] | 0.12 | 0.68 [0.43,1.09] | 0.11 |
|  | Self-Reported Preference for Women | Nonbinary/  genderqueer | 1.73 [1.1,2.73] | 0.02 | 1.81 [1.14,2.87] | 0.01 | 1.83 [1.15,2.9] | 0.01 | 1.66 [1.01,2.73] | 0.05 | 1.74 [1.05,2.88] | 0.03 | 1.76 [1.06,2.92] | 0.03 |
|  | Self-Reported Experiences of Traditional Sexism [ref: 0] | Man | 0.88 [0.44,1.74] | 0.71 | 0.72 [0.35,1.48] | 0.37 | 0.72 [0.35,1.48] | 0.37 | 0.86 [0.43,1.71] | 0.66 | 0.7 [0.34,1.44] | 0.33 | 0.69 [0.33,1.44] | 0.32 |
|  | Self-Reported Experiences of Traditional Sexism [ref: 0] | Nonbinary/  genderqueer | 0.75 [0.34,1.68] | 0.49 | 0.72 [0.31,1.65] | 0.43 | 0.69 [0.29,1.61] | 0.39 | 0.78 [0.33,1.89] | 0.59 | 0.74 [0.3,1.82] | 0.51 | 0.72 [0.29,1.81] | 0.48 |
|  | Self-Reported Experiences of Traditional Sexism [ref: 0] | Woman | 1.09 [0.42,2.86] | 0.86 | 1.09 [0.4,2.94] | 0.87 | 1.08 [0.4,2.9] | 0.89 | 0.82 [0.29,2.31] | 0.71 | 0.78 [0.27,2.24] | 0.64 | 0.77 [0.27,2.22] | 0.63 |
| Heterosexism |  |  |  |  |  |  |  |  |  |  |  |  |  |  |
|  | Implicit Recognition of Heterosexism toward LGBQ individuals | Straight | 0.98 [0.57,1.68] | 0.93 | 1.04 [0.62,1.75] | 0.88 | 1.05 [0.61,1.8] | 0.86 | 0.98 [0.56,1.73] | 0.95 | 0.99 [0.57,1.73] | 0.98 | 1 [0.57,1.77] | 0.99 |
|  | Implicit Recognition of Heterosexism toward LGBQ individuals | LGBQ | 1.09 [0.83,1.43] | 0.53 | 1.11 [0.84,1.46] | 0.47 | 1.11 [0.84,1.46] | 0.47 | 1.06 [0.79,1.42] | 0.68 | 1.1 [0.81,1.48] | 0.54 | 1.1 [0.81,1.48] | 0.54 |
|  | Internalized Preference for LGBQ individuals | Straight | 1.11 [0.67,1.83] | 0.69 | 1.24 [0.73,2.09] | 0.42 | 1.28 [0.74,2.21] | 0.38 | 1.16 [0.66,2.04] | 0.6 | 1.29 [0.72,2.3] | 0.4 | 1.31 [0.73,2.37] | 0.37 |
|  | Internalized Preference for LGBQ individuals | LGBQ | 1.07 [0.81,1.42] | 0.61 | 1.06 [0.79,1.42] | 0.69 | 1.06 [0.79,1.42] | 0.7 | 1.02 [0.75,1.38] | 0.91 | 0.99 [0.72,1.36] | 0.95 | 0.99 [0.72,1.36] | 0.95 |
|  | Self-Reported Recognition of Heterosexism toward LGBQ individuals | Straight | 1.36 [0.9,2.07] | 0.14 | 1.32 [0.87,2] | 0.19 | 1.33 [0.88,2.02] | 0.18 | 1.31 [0.86,2] | 0.21 | 1.28 [0.84,1.95] | 0.24 | 1.3 [0.86,1.98] | 0.22 |
|  | Self-Reported Recognition of Heterosexism toward LGBQ individuals | LGBQ | 1.11 [0.84,1.46] | 0.45 | 1.01 [0.77,1.32] | 0.96 | 1.01 [0.77,1.33] | 0.94 | 1.07 [0.81,1.43] | 0.63 | 0.96 [0.72,1.29] | 0.8 | 0.97 [0.72,1.3] | 0.83 |
|  | Self-Reported Preference for LGBQ individuals | Straight | 0.75 [0.47,1.2] | 0.23 | 0.85 [0.53,1.37] | 0.51 | 0.89 [0.54,1.46] | 0.64 | 0.75 [0.45,1.25] | 0.27 | 0.85 [0.51,1.42] | 0.53 | 0.9 [0.53,1.55] | 0.71 |
|  | Self-Reported Preference for LGBQ individuals | LGBQ | 1.15 [0.86,1.55] | 0.34 | 1.14 [0.85,1.54] | 0.39 | 1.14 [0.84,1.54] | 0.39 | 1.12 [0.82,1.52] | 0.48 | 1.12 [0.82,1.55] | 0.48 | 1.13 [0.82,1.56] | 0.46 |
|  | Self-Reported Experiences of Heterosexism [ref: 0] | Straight | 1.57 [0.54,4.56] | 0.4 | 1.04 [0.33,3.25] | 0.94 | 1.04 [0.33,3.27] | 0.95 | 1.41 [0.47,4.24] | 0.55 | 0.92 [0.28,2.96] | 0.88 | 0.93 [0.29,3] | 0.9 |
|  | Self-Reported Experiences of Heterosexism [ref: 0] | LGBQ | 1.26 [0.66,2.41] | 0.47 | 1.22 [0.63,2.38] | 0.55 | 1.22 [0.63,2.38] | 0.56 | 1.16 [0.59,2.26] | 0.67 | 1.17 [0.58,2.37] | 0.65 | 1.17 [0.58,2.36] | 0.66 |
| Cissexism |  |  |  |  |  |  |  |  |  |  |  |  |  |  |
|  | Implicit Recognition of Cissexism toward Non-cisgender individuals | Cisgender | 1.07 [0.8,1.41] | 0.66 | 1.14 [0.85,1.52] | 0.39 | 1.13 [0.84,1.52] | 0.41 | 1.1 [0.82,1.49] | 0.52 | 1.14 [0.84,1.56] | 0.4 | 1.15 [0.84,1.57] | 0.38 |
|  | Implicit Recognition of Cissexism toward Non-cisgender individuals | Not cisgender | 1.02 [0.7,1.48] | 0.93 | 1.12 [0.76,1.65] | 0.57 | 1.11 [0.75,1.64] | 0.6 | 1.1 [0.72,1.66] | 0.67 | 1.16 [0.75,1.78] | 0.5 | 1.15 [0.75,1.77] | 0.52 |
|  | Internalized Preference for Non-cisgender individuals | Cisgender | 0.83 [0.6,1.15] | 0.27 | 0.88 [0.63,1.23] | 0.46 | 0.87 [0.62,1.22] | 0.41 | 0.75 [0.52,1.08] | 0.12 | 0.79 [0.54,1.14] | 0.21 | 0.78 [0.54,1.13] | 0.19 |
|  | Internalized Preference for Non-cisgender individuals | Not cisgender | 0.88 [0.64,1.2] | 0.41 | 0.93 [0.67,1.29] | 0.66 | 0.91 [0.65,1.28] | 0.59 | 0.81 [0.55,1.17] | 0.26 | 0.85 [0.58,1.25] | 0.4 | 0.84 [0.57,1.24] | 0.37 |
|  | Self-Reported Recognition of Cissexism toward Non-cisgender individuals | Cisgender | 1.18 [0.87,1.59] | 0.28 | 1.21 [0.88,1.65] | 0.23 | 1.21 [0.88,1.68] | 0.25 | 1.15 [0.84,1.56] | 0.39 | 1.16 [0.84,1.61] | 0.35 | 1.18 [0.84,1.64] | 0.34 |
|  | Self-Reported Recognition of Cissexism toward Non-cisgender individuals | Not cisgender | 1.09 [0.72,1.64] | 0.69 | 1.1 [0.72,1.66] | 0.67 | 1.11 [0.73,1.69] | 0.62 | 1.15 [0.74,1.79] | 0.54 | 1.15 [0.73,1.8] | 0.56 | 1.17 [0.75,1.84] | 0.49 |
|  | Self-Reported Preference for Non-cisgender individuals | Cisgender | 1.43 [0.99,2.05] | 0.05 | 1.47 [1.02,2.12] | 0.04 | 1.52 [1.04,2.21] | 0.03 | 1.36 [0.95,1.96] | 0.09 | 1.4 [0.97,2.03] | 0.07 | 1.46 [1,2.13] | 0.05 |
|  | Self-Reported Preference for Non-cisgender individuals | Not cisgender | 1.09 [0.8,1.49] | 0.58 | 1.09 [0.79,1.5] | 0.6 | 1.11 [0.8,1.54] | 0.52 | 1.21 [0.86,1.72] | 0.28 | 1.19 [0.82,1.71] | 0.36 | 1.22 [0.84,1.76] | 0.29 |
|  | Self-Reported Experiences of Cissexism [ref: 0] | Cisgender | 2.18 [1.27,3.75] | 0 | 1.97 [1.12,3.46] | 0.02 | 1.96 [1.11,3.45] | 0.02 | 2.01 [1.16,3.51] | 0.01 | 1.83 [1.03,3.25] | 0.04 | 1.84 [1.03,3.27] | 0.04 |
|  | Self-Reported Experiences of Cissexism [ref: 0] | Not cisgender | 0.66 [0.27,1.59] | 0.35 | 0.65 [0.26,1.63] | 0.36 | 0.64 [0.25,1.63] | 0.35 | 0.55 [0.21,1.44] | 0.22 | 0.54 [0.2,1.49] | 0.24 | 0.55 [0.2,1.51] | 0.24 |
| Ageism |  |  |  |  |  |  |  |  |  |  |  |  |  |  |
|  | Implicit Recognition of Ageism toward Older individuals | Younger | 0.9 [0.7,1.16] | 0.4 | 0.87 [0.67,1.13] | 0.28 | 0.87 [0.67,1.13] | 0.3 | 0.85 [0.65,1.11] | 0.23 | 0.8 [0.61,1.07] | 0.13 | 0.81 [0.61,1.07] | 0.14 |
|  | Implicit Recognition of Ageism toward Older individuals | Older | 0.91 [0.56,1.47] | 0.69 | 0.81 [0.5,1.33] | 0.41 | 0.82 [0.5,1.35] | 0.44 | 0.88 [0.53,1.44] | 0.6 | 0.8 [0.48,1.36] | 0.41 | 0.8 [0.48,1.36] | 0.41 |
|  | Internalized Preference for Older individuals | Younger | 1.12 [0.87,1.44] | 0.38 | 1.18 [0.9,1.53] | 0.23 | 1.18 [0.91,1.55] | 0.22 | 1.16 [0.89,1.52] | 0.27 | 1.24 [0.94,1.65] | 0.13 | 1.25 [0.94,1.65] | 0.13 |
|  | Internalized Preference for Older individuals | Older | 0.99 [0.64,1.53] | 0.96 | 0.98 [0.62,1.55] | 0.94 | 1 [0.63,1.58] | 1 | 1.03 [0.67,1.61] | 0.88 | 1.05 [0.65,1.68] | 0.85 | 1.07 [0.66,1.72] | 0.79 |
|  | Self-Reported Recognition of Ageism toward Older individuals | Younger | 1.27 [0.98,1.63] | 0.07 | 1.24 [0.97,1.6] | 0.09 | 1.25 [0.97,1.6] | 0.09 | 1.28 [0.99,1.65] | 0.06 | 1.27 [0.98,1.65] | 0.07 | 1.27 [0.98,1.65] | 0.07 |
|  | Self-Reported Recognition of Ageism toward Older individuals | Older | 0.72 [0.49,1.05] | 0.09 | 0.78 [0.53,1.15] | 0.2 | 0.77 [0.52,1.14] | 0.19 | 0.72 [0.48,1.06] | 0.1 | 0.77 [0.51,1.16] | 0.2 | 0.76 [0.5,1.15] | 0.2 |
|  | Self-Reported Preference for Older individuals | Younger | 1.09 [0.85,1.39] | 0.52 | 1.03 [0.81,1.32] | 0.79 | 1.03 [0.81,1.32] | 0.79 | 1.06 [0.82,1.37] | 0.63 | 1.01 [0.78,1.31] | 0.93 | 1.01 [0.78,1.31] | 0.92 |
|  | Self-Reported Preference for Older individuals | Older | 1.26 [0.8,1.98] | 0.32 | 1.05 [0.66,1.65] | 0.85 | 1.07 [0.67,1.7] | 0.77 | 1.2 [0.76,1.9] | 0.43 | 1.02 [0.64,1.64] | 0.92 | 1.05 [0.65,1.7] | 0.84 |
|  | Self-Reported Experiences of Ageism [ref: 0] | Younger | 0.95 [0.59,1.53] | 0.83 | 0.86 [0.52,1.41] | 0.55 | 0.86 [0.52,1.41] | 0.55 | 0.9 [0.55,1.47] | 0.68 | 0.83 [0.5,1.37] | 0.46 | 0.83 [0.5,1.37] | 0.47 |
|  | Self-Reported Experiences of Ageism [ref: 0] | Older | 1.28 [0.56,2.91] | 0.56 | 1.06 [0.45,2.5] | 0.89 | 1.03 [0.44,2.44] | 0.94 | 1.5 [0.64,3.54] | 0.35 | 1.19 [0.48,2.97] | 0.7 | 1.17 [0.47,2.92] | 0.73 |
| Sizeism |  |  |  |  |  |  |  |  |  |  |  |  |  |  |
|  | Implicit Recognition of Sizeism toward Heavier individuals | Not Obese | 0.94 [0.72,1.23] | 0.65 | 0.91 [0.69,1.19] | 0.47 | 0.91 [0.69,1.19] | 0.48 | 0.91 [0.69,1.2] | 0.52 | 0.89 [0.67,1.17] | 0.41 | 0.89 [0.67,1.17] | 0.41 |
|  | Implicit Recognition of Sizeism toward Heavier individuals | Obese | 1.09 [0.74,1.6] | 0.67 | 1.09 [0.73,1.63] | 0.67 | 1.09 [0.73,1.64] | 0.66 | 1.15 [0.76,1.73] | 0.52 | 1.13 [0.73,1.74] | 0.59 | 1.13 [0.73,1.74] | 0.59 |
|  | Internalized Preference for Heavier individuals | Not Obese | 0.95 [0.73,1.25] | 0.74 | 0.89 [0.67,1.18] | 0.41 | 0.89 [0.67,1.18] | 0.41 | 0.91 [0.69,1.19] | 0.48 | 0.87 [0.65,1.17] | 0.36 | 0.88 [0.65,1.17] | 0.37 |
|  | Internalized Preference for Heavier individuals | Obese | 0.56 [0.37,0.86] | 0.01 | 0.54 [0.35,0.85] | 0.01 | 0.54 [0.34,0.85] | 0.01 | 0.49 [0.31,0.79] | 0 | 0.49 [0.3,0.8] | 0 | 0.49 [0.29,0.8] | 0.01 |
|  | Self-Reported Recognition of Sizeism toward Heavier individuals | Not Obese | 0.96 [0.74,1.23] | 0.74 | 0.89 [0.69,1.15] | 0.36 | 0.88 [0.68,1.15] | 0.36 | 0.94 [0.73,1.21] | 0.61 | 0.87 [0.67,1.12] | 0.28 | 0.86 [0.66,1.12] | 0.26 |
|  | Self-Reported Recognition of Sizeism toward Heavier individuals | Obese | 0.95 [0.66,1.38] | 0.79 | 0.88 [0.6,1.28] | 0.5 | 0.89 [0.6,1.31] | 0.54 | 0.91 [0.61,1.37] | 0.65 | 0.84 [0.55,1.29] | 0.44 | 0.86 [0.56,1.32] | 0.48 |
|  | Self-Reported Preference for Heavier individuals | Not obese | 1.37 [1.02,1.83] | 0.03 | 1.17 [0.87,1.59] | 0.29 | 1.18 [0.87,1.6] | 0.29 | 1.39 [1.03,1.87] | 0.03 | 1.21 [0.89,1.64] | 0.23 | 1.21 [0.89,1.66] | 0.22 |
|  | Self-Reported Preference for Heavier individuals | Obese | 1.09 [0.77,1.55] | 0.62 | 1.04 [0.72,1.5] | 0.83 | 1.04 [0.72,1.5] | 0.83 | 1.33 [0.9,1.96] | 0.15 | 1.24 [0.83,1.84] | 0.29 | 1.24 [0.83,1.85] | 0.29 |
|  | Self-Reported Experiences of Sizeism [ref: 0] | Not Obese | 1.52 [0.88,2.61] | 0.13 | 1.4 [0.79,2.46] | 0.25 | 1.42 [0.8,2.52] | 0.23 | 1.45 [0.84,2.51] | 0.18 | 1.38 [0.78,2.46] | 0.27 | 1.41 [0.79,2.52] | 0.24 |
|  | Self-Reported Experiences of Sizeism [ref: 0] | Obese | 1.25 [0.59,2.63] | 0.56 | 1.29 [0.6,2.79] | 0.52 | 1.33 [0.61,2.91] | 0.48 | 1.38 [0.62,3.09] | 0.43 | 1.52 [0.66,3.52] | 0.33 | 1.53 [0.65,3.59] | 0.32 |
|  | Implicit Recognition of Sizeism toward Heavier individuals | Not Obese | 0.94 [0.72,1.23] | 0.65 | 0.91 [0.69,1.19] | 0.47 | 0.91 [0.69,1.19] | 0.48 | 0.91 [0.69,1.2] | 0.52 | 0.89 [0.67,1.17] | 0.41 | 0.89 [0.67,1.17] | 0.41 |
|  | Implicit Recognition of Sizeism toward Heavier individuals | Obese | 1.09 [0.74,1.6] | 0.67 | 1.09 [0.73,1.63] | 0.67 | 1.09 [0.73,1.64] | 0.66 | 1.15 [0.76,1.73] | 0.52 | 1.13 [0.73,1.74] | 0.59 | 1.13 [0.73,1.74] | 0.59 |
|  | Internalized Preference for Heavier individuals | Not Obese | 0.95 [0.73,1.25] | 0.74 | 0.89 [0.67,1.18] | 0.41 | 0.89 [0.67,1.18] | 0.41 | 0.91 [0.69,1.19] | 0.48 | 0.87 [0.65,1.17] | 0.36 | 0.88 [0.65,1.17] | 0.37 |
|  | Internalized Preference for Heavier individuals | Obese | 0.56 [0.37,0.86] | 0.01 | 0.54 [0.35,0.85] | 0.01 | 0.54 [0.34,0.85] | 0.01 | 0.49 [0.31,0.79] | 0 | 0.49 [0.3,0.8] | 0 | 0.49 [0.29,0.8] | 0.01 |
|  | Self-Reported Recognition of Sizeism toward Heavier individuals | Not Obese | 0.96 [0.74,1.23] | 0.74 | 0.89 [0.69,1.15] | 0.36 | 0.88 [0.68,1.15] | 0.36 | 0.94 [0.73,1.21] | 0.61 | 0.87 [0.67,1.12] | 0.28 | 0.86 [0.66,1.12] | 0.26 |
|  | Self-Reported Recognition of Sizeism toward Heavier individuals | Obese | 0.95 [0.66,1.38] | 0.79 | 0.88 [0.6,1.28] | 0.5 | 0.89 [0.6,1.31] | 0.54 | 0.91 [0.61,1.37] | 0.65 | 0.84 [0.55,1.29] | 0.44 | 0.86 [0.56,1.32] | 0.48 |

**Appendix D: Supplemental Table 3. Random-effects meta-regression analysis: Associations of implicit (B-IAT) and explicit exposures with smoking/vaping in Life + Health Study participants (US-Born ages 25-64 years recruited from 3 community health centers), Boston, Massachusetts, 2020-2022.**

| **Interaction Model**   \| **Term** \| **Beta**  **estimate+** \| **Standard**  **error** \| **Lower bound** \| **Upper**  **bound** \| **p-value** \| \| --- \| --- \| --- \| --- \| --- \| --- \| \| Intercept \| -0.06574 \| 0.089983 \| -0.24210 \| 0.110626 \| 0.465053 \| \| **Explicit/Implicit Discrimination and Target/Dominant Groups** \|  \|  \|  \|  \|  \| \| Explicit vs. Implicit \| 0.162725 \| 0.085325 \| -0.00451 \| 0.329959 \| 0.056504 \| \| Interaction Explicit vs. Implicit x Target vs. Dominant \| -0.1654 \| 0.124305 \| -0.40903 \| 0.078235 \| 0.183326 \| \| Target vs. Dominant \| 0.01953 \| 0.093677 \| -0.16407 \| 0.203132 \| 0.834855 \| \| **Domains** \|  \|  \|  \|  \|  \| \| Racism \| 0.084528 \| 0.106492 \| -0.12419 \| 0.293249 \| 0.427342 \| \| Sexism \| 0.065206 \| 0.10357 \| -0.13779 \| 0.268199 \| 0.528964 \| \| Heterosexism \| 0.091275 \| 0.111425 \| -0.12711 \| 0.309664 \| 0.412695 \| \| Cissexism \| 0.118533 \| 0.107407 \| -0.09198 \| 0.329047 \| 0.269770 \| \| Ageism \| Ref \| -- \| -- \| -- \| -- \| \| Sizeism \| -0.03449 \| 0.104454 \| -0.23922 \| 0.170235 \| 0.741247 \|   +Additive Scale: Estimates are on the log odds scale and represent the additive change in the log odds of current smoking/vaping.  To obtain multiplicative scale estimates, additive estimates can be exponentiated and will represent the change in the odds of current smoking/vaping.  Ageism is the referent group for the domain variable. For example, there is a 13% greater odds of smoking/vaping comparing a 1 standard deviation change  in cissexism measures compared to a one standard deviation change in the ageism measures [(exp(0.118533)]. |
| --- | --- | --- | --- | --- | --- | --- | --- | --- | --- | --- | --- | --- | --- | --- | --- | --- | --- | --- | --- | --- | --- | --- | --- | --- | --- | --- | --- | --- | --- | --- | --- | --- | --- | --- | --- | --- | --- | --- | --- | --- | --- | --- | --- | --- | --- | --- | --- | --- | --- | --- | --- | --- | --- | --- | --- | --- | --- | --- | --- | --- | --- | --- | --- | --- | --- | --- | --- | --- | --- | --- | --- | --- | --- | --- | --- | --- | --- | --- |
